# Interleukin-30 subverts prostate cancer-endothelium crosstalk by fostering angiogenesis and activating immunoregulatory and oncogenic signaling pathways

**DOI:** 10.1186/s13046-023-02902-y

**Published:** 2023-12-12

**Authors:** Stefania Livia Ciummo, Carlo Sorrentino, Cristiano Fieni, Emma Di Carlo

**Affiliations:** 1 Department of Medicine and Sciences of Aging, “G. d’Annunzio” University” of Chieti-Pescara, Chieti, Italy; 2 Anatomic Pathology and Immuno-Oncology Unit, Center for Advanced Studies and Technology (CAST), “G. d’Annunzio” University of Chieti-Pescara, Via L. Polacchi 11, 66100 Chieti, Italy

**Keywords:** Prostate Cancer, Endothelium, Interleukin-30, Angiogenesis stimulating factors, CRISPR/Cas9, Tumor microenvironment

## Abstract

**Background:**

Cancer-endothelial interplay is crucial for tumor behavior, yet the molecular mechanisms involved are largely unknown. Interleukin(IL)-30, which is expressed as a membrane-anchored cytokine by human prostate cancer (PC) cells, promotes PC vascularization and progression, but the underlying mechanisms have yet to be fully explored.

**Methods:**

PC-endothelial cell (EC) interactions were investigated, after coculture, by flow cytometry, transcriptional profiling, western blot, and ELISA assays. Proteome profiler phospho-kinase array unveiled the molecular pathways involved. The role of tumor-derived IL30 on the endothelium's capacity to generate autocrine circuits and vascular budding was determined following IL30 overexpression, by gene transfection, or its deletion by CRISPR/Cas9 genome editing. Clinical value of the experimental findings was determined through immunopathological study of experimental and patient-derived PC samples, and bioinformatics of gene expression profiles from PC patients.

**Results:**

Contact with PC cells favors EC proliferation and production of angiogenic and angiocrine factors, which are boosted by PC expression of IL30, that feeds autocrine loops, mediated by IGF1, EDN1, ANG and CXCL10, and promotes vascular budding and inflammation, via phosphorylation of multiple signaling proteins, such as Src, Yes, STAT3, STAT6, RSK1/2, c-Jun, AKT and, primarily CREB, GSK-3α/β, HSP60 and p53. Deletion of the IL30 gene in PC cells inhibits endothelial expression of IGF1, EDN1, ANG and CXCL10 and substantially impairs tumor angiogenesis. In its interaction with IL30-overexpressing PC cells the endothelium boosts their expression of a wide range of immunity regulatory genes, including CCL28, CCL4, CCL5, CCR2, CCR7, CXCR4, IL10, IL13, IL17A, FASLG, IDO1, KITLG, TNFA, TNFSF10 and PDCD1, and cancer driver genes, including BCL2, CCND2, EGR3, IL6, VEGFA, KLK3, PTGS1, LGALS4, GNRH1 and SHBG. Immunopathological analyses of PC xenografts and in silico investigation of 1116 PC cases, from the Prostate Cancer Transcriptome Atlas, confirmed the correlation between the expression of IL30 and that of both pro-inflammatory genes, NOS2, TNFA, CXCR5 and IL12B, and cancer driver genes, LGALS4, GNRH1 and SHBG, which was validated in a cohort of 80 PC patients.

**Conclusions:**

IL30 regulates the crosstalk between PC and EC and reshapes their transcriptional profiles, triggering angiogenic, immunoregulatory and oncogenic gene expression programs. These findings highlight the angiostatic and oncostatic efficacy of targeting IL30 to fight PC.

**Supplementary Information:**

The online version contains supplementary material available at 10.1186/s13046-023-02902-y.

## Background

Prostate cancer (PC) is the most common type of cancer, after breast cancer (BC), with 20.3 million new cases expected worldwide by 2030. Of the predicted patients, as many as 13.2 million will not survive, due to disease progression and metastasis [1]. Tumor progression is typically associated with an angiogenic switch, the process whereby the normal endothelium of the existing vasculature transits from a quiescent to an activated and proliferating state, which leads to the development of new blood vessels and fuels tumor growth [2]. Angiogenesis plays a major role in the development and spread of PC [3] and microvessel density (MVD) has been shown to be a predictor of metastasis in PC patients [4]. A wide range of growth factors, immune mediators and receptors are known to be involved in the cross communication between PC and endothelium, and to regulate leukocyte migration and activation, such as CCL2, CCL5, CXCR1 and CCR3 [5, 6], or tumor vascularization, such as matrix metalloproteinases (MMPs) [7], hypoxia inducible factor (HIF)-1 [8], vascular endothelial growth factor (VEGF), VEGF tyrosine kinase receptor (VEGFR)-1 [9, 10], transforming growth factor β (TGFβ) [11] and cyclooxygenase 2 (COX2) [12]. However, the panorama of molecular mechanisms primed by PC-endothelial network interactions, that shape the tumor microenvironment (TME) and behavior, remains largely unexplored and may provide novel, more suitable, therapeutic targets.

We recently demonstrated that the immunoregulatory molecule, interleukin(IL)-30 (IL27/p28) [13], which is expressed as a membrane-anchored cytokine, by human PC cells, and in the microenvironment, by tumor-infiltrating immune cells, mainly macrophages and myeloid-derived suppressor cells (MDSCs) [14, 15], plays a critical role in PC onset and progression, by triggering a cascade of proinflammatory and oncogenic events, in association with the development of a robust vascular network [16, 17]. A rich vascular supply has been described in IL30-overexpressing prostatic and mammary tumors, in both human and murine models of cancer, by contrast, a poor vascularization characterized the slow growing IL30-deficient tumors [15–18].

Here, we investigate the reciprocal contact-dependent regulation of the angiogenic, immunoregulatory and oncogenic programs in PC and endothelial cells (ECs), respectively, and highlight the impact of PC-derived IL30 on the genotypic and phenotypic profiles of ECs, but also the feedback on PC gene expression programs of the EC response to the tumor expression of IL30. Bioinformatics and immunopathological studies on clinical samples from independent cohorts of PC patients underline the translational value of the experimental findings and highlight the antiangiogenic implication of a therapeutic, tumor selective, IL30 inhibition to fight PC progression.

## Methods

### Study design

For mouse studies, sample sizes were determined by minimizing the number of animals essential to statistically significant results (in accordance with the 3R principles). An overall sample size of 15 mice per group allowed the detection of a statistically significant difference between the experimental groups, with an 80% power, at a 0.05 significance level. Animals were randomly assigned to study arms.

For studies on human tissue samples, a cohort of 80 patients allowed the detection of a statistically significant correlation between two genes, with an 85% power and a 5% significance level (G*Power, RRID:SCR_013726). PC patients who had not received immunosuppressive treatments, hormone- or radiotherapy, and were free from immune system diseases, were selected by matching for Gleason score with patients from the *Prostate Cancer Transcriptome Atlas (PCTA)* (Table 1).

**Table 1:**
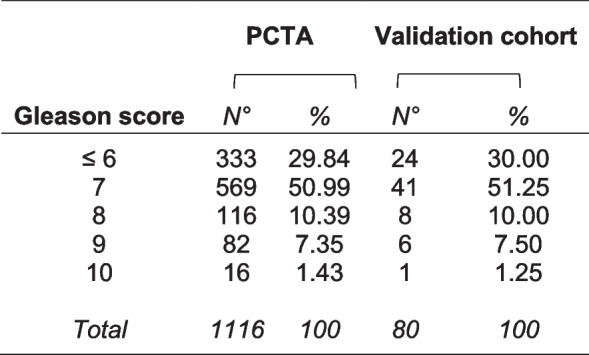
PC patients of the validation cohort matched by Gleason score with patients of the *PCTA* collection

All experiments were performed in blind. Study investigators were unaware to which group a particular animal was assigned to, and if a particular human PC sample was IL30 negative or positive.

### Cell culture and MTT assay

Primary human umbilical vein endothelial cells (HUVEC; #PCS-100–010) and immortalized human aortic endothelial cells (TeloHAEC; RRID:CVCL_Z065) were purchased from the American Type Culture Collection (ATCC, Manassas, VA, USA) and were cultivated in Vascular Cell Basal Medium (#PCS-100–030; ATCC) plus Endothelial Cell Growth Kit-VEGF (#PCS-100–041; ATCC). Human PC cell lines, AR^**−**^ PC3 and AR^**+**^ DU145 [15, 19, 20], were purchased from the ATCC, which authenticated them by short tandem repeat profile analysis. PC cells were cultured in RPMI-1640 (#R8758; Merck, Darmstadt, Germany) with 10% fetal calf serum (#F1283; Merck).

All cell lines were passaged for fewer than 6 months after resuscitation and were confirmed mycoplasma-free by PCR analysis**.**

For PC-Endothelial cell (EC) cocultures, PC and ECs, were seeded in 1:1 ratio and cultivated in Vascular Cell Basal Medium (ATCC; #PCS-100–030) plus Endothelial Cell Growth Kit-VEGF (ATCC; #PCS-100–041). Cell viability and proliferation were assessed using the CellTiter 96 AQueous One Solution Cell Proliferation Assay (#G3582; Promega, Madison, WI, USA), according to manufacturer’s instructions.

### PCR array and real-time RT-PCR

Real-time RT-PCR and PCR array were performed, as described in the Supplementary Methods, using the RT^2^ Profiler Human Angiogenesis PCR Array (#PAHS-024ZR)**,** RT^2^ Profiler Human Cancer Inflammation & Immunity Crosstalk PCR Array (#PAHS-181Z) and the RT^2^ Profiler™ Human Prostate Cancer PCR Array (#PAHS-135Z) (all from Qiagen, Hilden, Germany).

### Endothelial cell proliferation assay

To investigate the effect of IL30, produced by tumor cells, on endothelial cell proliferation, excluding the effect of other cytokines or soluble factors present in the extracellular matrix, we used the Growth Factor Reduced Matrigel (GFR-Matrigel), a soluble basement membrane extract, which contains the same growth factors present in standard Matrigel but in reduced concentrations. Briefly, the GFR-Matrigel (#354,230; BD Biosciences, Franklin Lakes, NJ, USA) was thawed on ice, at + 4 °C overnight, and then plated (125 µl/well) in 8-well chamber slides, which were incubated, at 37 °C for 30 min, to allow the Matrigel to polymerize [21]*.* Subsequently, 2 × 10^5^ HUVECs or HAECs were mixed with 2 × 10^5^ IL30-overexpressing DU145 or PC3, IL30KO-DU145 or -PC3, or the respective control cell lines, and added to the top of the Matrigel in each well. The chambers were then incubated at 37 °C for 24 h, fixed with acetone and stained as described in the section *Histology, immunohistochemistry and morphometric analyses.*

Endothelial cell counts were assessed by confocal microscopy, using a LSM 800 confocal microscope (Zeiss, Oberkochen, Germany; RRID:SCR_015963) and the ZEN Microscopy Software (Zeiss; RRID:SCR_013672). Four to six high-power fields were analyzed for each well and two optical sections per well were evaluated. Results were expressed as mean ± SD of CD31^+^cells per field.

### Endothelial tube formation assay

After thawing, GFR-Matrigel (BD Biosciences) was added (125 µl/well) into 8-well plates and allowed to polymerize at 37 °C for 30 min. Meanwhile, HUVEC or HAEC were detached from flasks and re-suspended in culture medium with or without rIL30 (50 ng/ml). Subsequently, 2 × 10^5^ HUVEC or HAEC were added to the top of the Matrigel in each well. The slides were then incubated at 37° for 24 h and the tube formation was evaluated, after CD31 immunofluorescence staining, by confocal microscopy, using the LSM 800 confocal microscope and the ZEN Microscopy Software (both from Zeiss). Capillaries were identified as small tubes or circles marked by CD31 staining. Four to six high-power fields were analyzed for each well and two optical sections per well were evaluated. Results were expressed as mean ± SD of CD31^+^capillaries/field.

### Flow cytometry

To assess phenotype markers and investigate surface expression of cytokines, cytokine receptors, growth factor receptors and adhesion molecules, human ECs were harvested and mechanically dissociated into a single cell suspension. Then, the cells were pelleted, resuspended in PBS and incubated for 30 min, at 4 °C, with the antibodies (Abs) listed in the Supplementary Table S1. Acquisition was performed using a BD Scientific Canto II Flow Cytometer (RRID:SCR_018056; BD Biosciences) and the data were analyzed using FlowJo software (RRID:SCR_008520; BD Biosciences). Dead cells were excluded by 7AAD staining. All experiments were performed in triplicate. To isolate PC and ECs for molecular studies, after their coculture, fluorescence-activated cell sorting (FACS) analyses were performed, using a BD FACS Aria II Cell Sorter (RRID:SCR_018091; BD Biosciences), as described in the Supplementary Methods.

### Transfection with *IL27p28* (*IL30*) expressing vector

For the overexpression of human *IL30* in DU145 and PC3 cells, we used the *IL27p28* Human Tagged ORF Clone (#RC209337L1; Origene, Rockville, MD, USA) which was transfected in cancer cells using Lipofectamine 3000 Reagent (#L3000001; Thermo Fisher Scientific, Waltham, MA, USA) as we described in ref. 15. The expression of IL30 was confirmed by real-time RT-PCR and western blot (WB) [15].

### CRISPR/Cas9-mediated *IL30* gene knockout

The CRISPR/Cas9 technology was used to generate *IL30* gene knockout (IL30KO) DU145 and PC3 cells, as we described in ref.15. IL30 gene knockout was validated by WB [15].

### ELISA

Quantitation of ANG, CXCL10, EDN1 and IGF1, in the supernatant derived from human endothelial cells, was carried out using the following ELISA kits, according to manufacturer’s instructions: Angiogenin Human ELISA Kit (#EHANG); CXCL10 Human ELISA Kit (#KAC2361); Endothelin-1 Human ELISA Kit (#EIAET1) (all from Thermo Fisher Scientific, Waltham, MA, USA) and Human IGF1 ELISA Kit (#ab211651, Abcam, Cambridge, UK).

### Western blot

WB was performed, as described in the Supplementary Methods, to assess IL30, EBI3, ANG, CXCL9, EDN1, TGFB2, CXCL6, THBS2 and IGF1 protein expression in human endothelial cells, and IL1β, IL4, IL6, EGF, VEGFA, LGALS4 and SHBG protein expression in human PC cells.

### Human phospho-kinase antibody array

The Proteome Profiler Human Phospho-Kinase Array (#ARY003B; R&D Systems, Minneapolis, MN, USA) was used according to manufacturer’s instructions. Briefly, cells were lysed in manufacturer’s buffer, protein were quantified by Bradford Protein Assay (Bio-Rad, Hercules, CA, USA) and samples adjusted to 800 μg/ml with lysis buffer. Then, 334 μl of lysate was loaded per membrane and signals were detected by Chemi-Reagent Mix. The signal intensity of each spot was determined by ImageJ software (RRID:SCR_003070) and results were expressed as mean ± SD of pixel density. Reference spots were used to normalize signals across membranes. All experiments were performed in triplicate.

### Prostate cancer xenograft samples

NSG mice (RRID:IMSR_JAX:005557) were purchased from Charles River Laboratories (Wilmington, MA, USA). NSG mice were housed under high barrier conditions, according to the Jackson Laboratory’s guidelines, in the animal facility of the Center for Advanced Studies and Technology, Chieti, Italy.

To study in vivo the effects of IL30 overexpression or knockout, in PC cells, on tumor vasculature and expression of genes driving angiogenesis, inflammation and prostatic carcinogenesis, three groups (15 mice per group) of 8-week-old NSG mice were subcutaneously injected with 3 × 10^5^ wild-type (CTRL), Empty Vector (EV) or hIL30 lentiviral-DNA (IL30LV-DNA) transfected DU145 or PC3 cells, and another three groups of fifteen 8-week-old NSG mice, with 5 × 10^5^ wild-type (CTRL), non-targeting guide RNA-treated (NTgRNA) or IL30 knockout (IL30KO) DU145 or PC3 cells. Tumors were measured with calipers as soon as they were palpable, and mice were sacrificed when the tumor reached 700 mm^3^, since at this size there are still no important necrotic phenomena that can invalidate the immunohistochemical examination. An overall sample size of 15 mice per group allowed the detection of a statistically significant difference between the three groups, with an 80% power, at a 0.05 significance level (G*Power, RRID:SCR_013726).

Animal procedures were performed in accordance with the European Community and ARRIVE guidelines and were approved by the Institutional Animal Care Committee of “G. d’Annunzio” University and by the Italian Ministry of Health (Authorization n. 892/2018-PR).

### Bioinformatic analyses

For bioinformatic analyses, we used the “*Prostate Cancer Transcriptome Atlas (PCTA)*”, the largest of the publicly available online databases, which includes RNA-Seq data from 1116 clinical PC specimens, with annotation of Gleason score, collected from 38 PC datasets and normalized by median centering method and quantile scaling (http://www.thepcta.org* and ref 47*). We used the resulting merged database to measure the statistical correlation between pairs of genes or between single genes and the apoptotic signaling pathway dataset reported in the *PCTA* (Supplementary Table S2). All statistical analyses were performed by applying the Spearman's rank correlation coefficient (*ρ*) calculation tool included in the *PCTA* website, at an α level of 0.05.

### Patients and samples

Tissue samples were collected and stored in the institutional Biobank of the Local Health Authority n. 2 Lanciano Vasto Chieti (Italy) and the personal data processing complies with Data Protection Laws. For this study, we selected, by matching for Gleason score with patients from the *PCTA* (Table 1), a validation cohort of 80 patients, who underwent radical prostatectomy for PC and had not received immunosuppressive treatments, hormone- or radiotherapy, and were free from immune system diseases. This sample size allowed the detection of a statistically significant correlation between two genes, with an 85% power and a 5% significance level (G*Power, RRID:SCR_013726). The study was approved by the Ethical Committee of the “G. d’Annunzio” University and Local Health Authority of Chieti (PROT. 1945/09 COET of 14/07/2009, amended in 2012), and was performed, after written informed consent from patients, in accordance with the principles outlined in the Declaration of Helsinki.

### Histology, immunohistochemistry and morphometric analyses

For histology, tissue samples were fixed in 4% formalin, embedded in paraffin, sectioned at 4 μm and stained with hematoxylin and eosin. Immunofluorescent stainings for CD31 and EpCAM and immunostainings for CD31, Ki67, CXCR5, EpCAM, IL12β, IL30, IGF1, LGALS4, NOS2, SHBG, TGFα and TNFα, were performed as described in *ref. 18,* using the Abs listed in the Supplementary Table S3. Proliferation index, microvessel counts and expression of immunoregulatory and prostate cancer driver genes in tumor samples were assessed as described in the Supplementary Methods. The morphometric analysis, on single immunostained sections, was performed by light microscopy with a Leica Imaging Workstation and QWin image analysis software (Leica QWin, RRID:SCR_018940). The Spearman's rank correlation coefficients for each pair of markers were calculated using Stata V.13 (StataCorp, College Station, TX, USA; RRID:SCR_012763).

### Statistical analysis

For in vitro studies, in vivo immunohistochemical analyses on tumor xenografts and on human PC samples, for which data are approximately normally distributed, between-group differences were assessed by Student’s *t*-test, or ANOVA followed by Tukey HSD test. Spearman's correlation coefficient (*ρ*) was used to analyse correlations between the expression of IL30 and that of molecules resulting from bioinformatic findings. For the bioinformatics, statistical analyses have been described above.

All statistical tests were two-sided and evaluated at an α level of 0.05 using Stata V.13 (StataCorp, College Station, TX, USA; RRID:SCR_012763).

### Data availability

The data generated in this study are available upon request from the corresponding author. Expression profile data analyzed in this study were obtained from the *Prostate Cancer Transcriptome Atlas (PCTA)* collection, at http://www.thepcta.org.

## Results

### Contact with PC cells promotes EC proliferation and capillary bud formation, which are fostered by IL30 overexpression and suppressed by IL30 gene deletion in PC cells

Primarily found in high-grade and stage of PC, IL30 expression is associated with a thriving vasculature, immune suppression, and tumor progression [14–16]. To assess its potential impact on the PC-EC crosstalk, we used two human cell lines derived from the metastases of high-grade PCs, and that express membrane-anchored IL30 [15], namely DU145 cells [19], endowed with a CK8/14^+^AR^+^PSA^+^ phenotype, and castration resistant PC3 cells [20], endowed with a CD44^+^AR^−^PSA^−^CgA^+^NSE^+^ neuroendocrine phenotype. Both cell lines were engineered to overexpress IL30, henceforth referred to as IL30-DU145 and IL30-PC3 cells or were knocked out (KO) for the IL30 gene by CRISPR/Cas9 genome editing, hereinafter referred to as IL30KO-DU145 and IL30KO-PC3 cells [15]. Since the tumor vascular bed stems from the activation and proliferation of normal pre-existing vessels, for the PC-EC coculture experiments, we used primary ECs derived from human umbilical vein, HUVEC, and immortalized ECs isolated from human aortic endothelia, TeloHAEC, hereinafter referred to as HAEC. Both cell types were authenticated by STR (Eurofins Genomics, Ebersberg, Germany) and surface staining for characteristic markers (Supplementary Fig. S1).

**Fig. 1:**
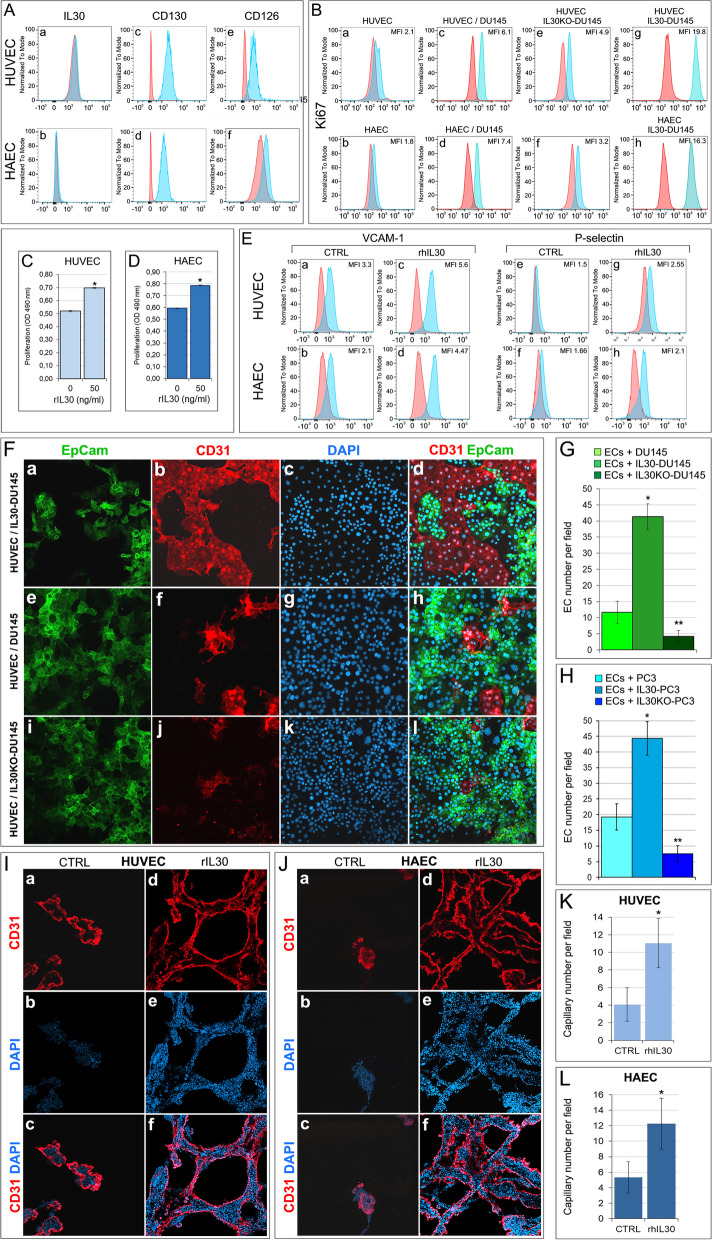
Interleukin-30 overexpression in PC cells stimulates endothelial cell proliferation and promotes vascular tube formation. **A** Cytofluorimetric analyses of IL30 (a, b), CD130 (c, d) and CD126 (e, f) expression in HUVEC top of the panel, and HAEC, bottom of the panel. Both EC types did not express IL30 but expressed the gp130 (CD130) and IL6Rα (CD126) receptor chains. Red lines: isotype control. Experiments were performed in triplicate. **B** Cytofluorimetric analyses of EC proliferation by Ki67 staining. When compared to ECs cultured alone (a, b), ECs cocultured with PC cells, DU145 (c, d), showed higher proliferation. Endothelial proliferation was suppressed by coculture with IL30KO-DU145 cells (e, f) and enhanced by coculture with IL30-DU145 cells (g, h). Red lines: isotype control. Experiments were performed in triplicate. **C**, **D** MTT assay of HUVEC (**C**) and HAEC (**D**), after 48 h of treatment with rhIL30 (50 ng/ml). *p < 0.0001, Student's t-test compared with untreated cells. Results are expressed as mean ± SD. Experiments were performed in triplicate. **E** Cytofluorimetric analyses of HUVEC (top of panel) and HAEC (bottom of panel) after the treatment with rhIL30 (50 ng/ml overnight and restimulation with 100 ng/ml for 4 h) revealed the upregulation of VCAM-1 (a-d) and P-selectin (e–h) compared to untreated (CTRL) cells. Experiments were performed in triplicate. **F** Confocal microscopy images of ECs (stained red with anti-CD31 Abs) cocultures with IL30-DU145 (a-d), wild type DU145 (e–h), or IL30KO-DU145 (i-l) cancer cells (stained green with anti-EpCAM Abs). Nuclei stained blue with DAPI. Representative images from three experiments. Magnification: X400. **G** Histograms representing the automated quantitative analysis, performed on confocal microscopy images, of the mean number ± SD of CD31^+^ECs after coculture with wild type, IL30-overexpressing or IL30 knockout DU145 (**G**) or PC3 (**H**) cells. Results are expressed as mean ± SD of CD31^+^cells per field. ANOVA: *p* < 0.0001. *p < 0.01, Tukey HSD Test compared with ECs + DU145 or PC3. **p < 0.01, Tukey HSD Test compared with ECs + DU145 or PC3 and ECs + IL30-DU145 or IL30-PC3. **I**, **J** Confocal microscopy images of capillary tube formation in Matrigel by red stained (anti-CD31 Abs) HUVEC (**I**) and HAEC (**J**) untreated (CTRL, a-c) or treated (50 ng/ml for 24 h, d-f) with rhIL30. Nuclei stained blue with DAPI. Representative images of three experiments. Magnification: X400. **K**, **L** Histograms representing the automated quantitative analysis, performed on confocal microscopy images, of the mean number ± SD of CD31^+^HUVEC (**F**) or CD31^+^HAEC (**G**) capillaries per field (X400), developed in Matrigel with or without (CTRL) addition of rhIL30. *p < 0.0001, Student's *t*-test *versus* CTRL. Experiments were performed in triplicate

Flow cytometric and western blot analysis demonstrated that HUVEC and HAEC did not express or produce IL30 (IL27/p28) (Fig. 1A, and Supplementary Fig. S2), but expressed CD130 (IL6R-beta) and CD126 (IL6R-alpha), which are currently known to function as the IL30 receptor (R) chains [22] (Fig. 1A).

Coculture with wild type PC cells, DU145 and PC3, increased (Student's *t*-test: *p* < 0.01) the proliferation of both HUVEC and HAEC, whereas deletion of the IL30 gene in PC cells dramatically decreased (Student's *t*-test: *p* < 0.01) EC proliferation, which was substantially improved by coculture with IL30 overexpressing PC cells (Student's *t*-test: *p* < 0.01), as shown by the flow cytometric analyses of Ki67 staining (Fig. 1B). EC apoptosis, as investigated by flow cytometric analyses of Annexin V staining, was unaffected by contact with wild type PC cells or with IL30-overexpressing or IL30-deleted PC cells (Supplementary Fig. S3). Moreover, treatment of both HUVEC and HAEC with recombinant human (rh) IL30 significantly (Student's *t*-test: *p* < 0.0001) fostered their proliferation, as shown by MTT assay (Fig. 1C, D), and activation, as demonstrated by flow cytometric analyses of adhesion molecule (ICAM1, ICAM2, VCAM1 and P-selectin) expression, showing the upregulation of VCAM1 (CD106) and P-selectin (CD162) (Fig. 1E), which mediate leukocyte adhesion and can shape the TME [23]. Notably, endothelial cell proliferation assay of HUVEC, cocultured with IL30-overexpressing or IL30 gene knockout DU145 and PC3 cells, confirmed that tumor-derived IL30 stimulates EC proliferation, which was suppressed by coculture with IL30KO-PC cells, as shown by confocal microscopy and automated quantitative image analysis of CD31^+^ECs cocultured with EpCAM^+^PC cells (Fig. 1F, G, H). Finally, endothelial cell tube formation assay revealed that the number of capillaries generated from HUVEC and HAEC after 24 h of culture in Matrigel embedded with rhIL30 was significantly higher than the number of capillaries developed in control cultures (Student's *t*-test: *p* < 0.0001) (Fig. 1I, J, K, L), therefore demonstrating IL30's ability to promote vascular budding.

### Contact with Interleukin-30 expressing PC cells reprograms the transcriptional profile of ECs and promotes autocrine angiogenic loops and in vivo tumor vascularization

Since contact with PC cells stimulated endothelial proliferation and capillary bud formation, which were further improved by their overexpression of IL30, and impoverished by IL30 gene deletion in PC cells, we wondered whether PC—EC contact, and membrane-anchored IL30 of PC cells, had an impact on the endothelial expression of angiogenesis-related genes.

**Fig. 2:**
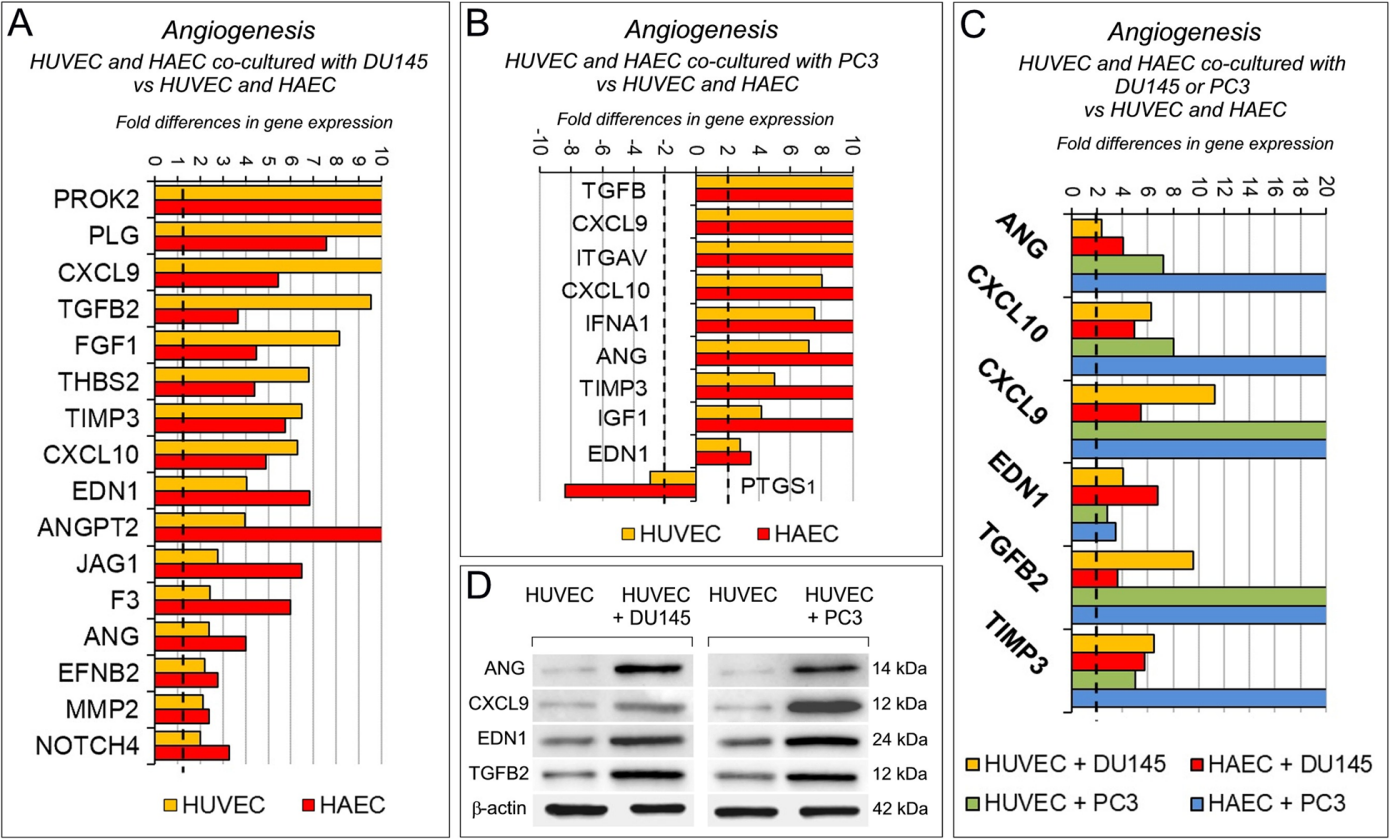
Contact with PC cells reshapes the transcriptional profile of endothelial cells. **A** Fold differences of mRNAs of angiogenesis-related genes, between HUVEC (yellow bars) or HAEC (red bars) cocultured with wild type DU145 *versus* HUVEC or HAEC, respectively. A significant threshold of a twofold change in gene expression corresponded to *p* < 0.001. Experiments were performed in duplicate. **B** Fold differences of mRNAs of angiogenesis-related genes between HUVEC (yellow bars) or HAEC (red bars) cocultured with wild type PC3 *versus* HUVEC or HAEC, respectively. A significant threshold of a twofold change in gene expression corresponded to *p* < 0.001. Experiments were performed in duplicate. **C** Fold differences of mRNAs of angiogenesis-related genes between HUVEC cocultured with wild type DU145 (yellow bars), or PC3 (green bars), *versus* HUVEC alone, and between HAEC cocultured with wild type DU145 (red bars), or PC3 (blue bars) *versus* HAEC alone. Genes whose regulation was shared by both endothelial cell types are represented. A significant threshold of a twofold change in gene expression corresponded to *p* < 0.001. Experiments were performed in duplicate. **D** Western blot analysis of ANG, CXCL9, EDN1 and TGFB2 protein expression in HUVEC cocultured with DU145 (left side of the panel) or PC3 cells (right side of the panel). Representative images of three experiments

Angiogenesis PCR array (Supplementary Table S4) revealed that coculture with PC cells substantially affects the transcriptional profile of ECs (Fig. 2A, B). Following coculture with DU145, both HUVEC and HAEC overexpressed *PROK2, PLG, CXCL9, TGFB2, FGF1, THBS2, TIMP3, CXCL10, EDN1, ANGPT2, JAG1, F3, ANG, EFNB2, MMP2, NOTCH4*. Coculture with PC3 cells led, in both EC types, to the upregulation of *TGFB2, CXCL9, ITGAV, CXCL10, IFNA1, ANG, TIMP3, IGF1* and *EDN1*, whereas only *PTGS1* was downregulated.

The upregulation of *ANG* [24], *CXCL10* [18], *CXCL9* [25], *EDN1* [26, 27], *TGFB2* [28] and *TIMP3* [29] was shared by both HUVEC and HAEC, after coculture with DU145 and PC3 cells (Fig. 2C). Therefore, contact with prostate cancer cells fostered endothelial expression of angiogenesis regulators, the majority with angiogenic effects, which was confirmed at protein level by western blot analyses (Fig. 2D, Supplementary Fig. S4).

**Fig. 3:**
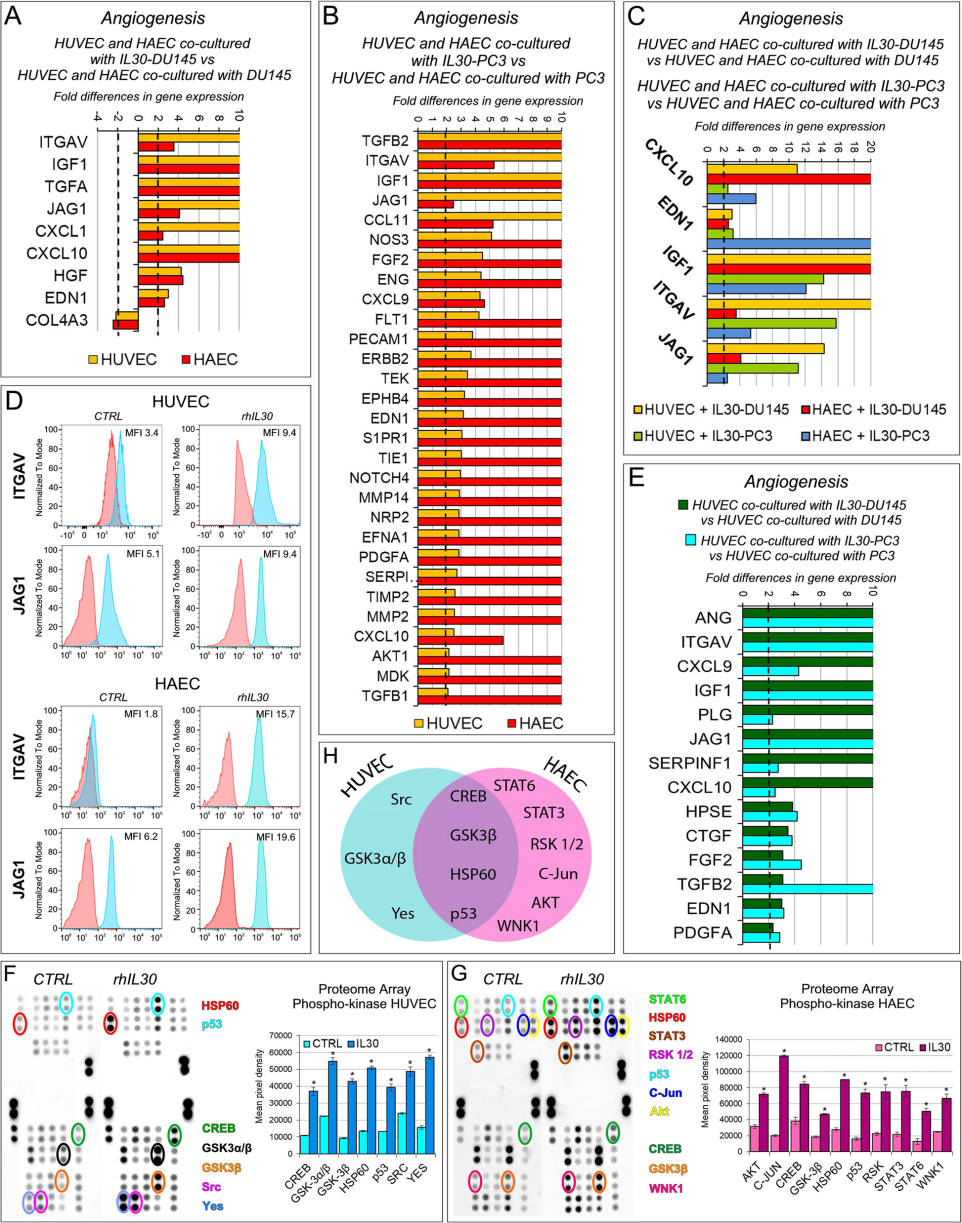
Contact with Interleukin-30 overexpressing PC cells leads to kinase-phosphorylation of multiple signaling proteins and expression of inflammation and angiogenesis-related genes in endothelial cells. **A** Fold differences of mRNAs of angiogenesis-related genes between HUVEC (yellow bars) or HAEC (red bars) cocultured with IL30-DU145 *versus* HUVEC, or HAEC, cocultured with wild type DU145. Results obtained from control EV-transfected cells were comparable to those from wild type cells. A significant threshold of a twofold change in gene expression corresponded to *p* < 0.001. Experiments were performed in duplicate. **B** Fold differences of mRNAs of angiogenesis-related genes between HUVEC (yellow bars) or HAEC (red bars) cocultured with IL30-PC3 *versus* HUVEC, or HAEC, cocultured with wild type PC3. Results obtained from control EV-transfected cells were comparable to those from wild type cells. A significant threshold of a twofold change in gene expression corresponded to *p* < 0.001. Experiments were performed in duplicate. **C** Fold differences of mRNAs of angiogenesis-related genes between HUVEC cocultured with IL30-DU145 (yellow bars), or IL30-PC3 (green bars), *versus* HUVEC cocultured with wild type DU145, or PC3, respectively. Fold differences of mRNAs of angiogenesis-related genes of HAEC cocultured with IL30-DU145 (red bars), or IL30-PC3 (blue bars) *versus* HAEC cocultured with wild type DU145, or PC3, respectively. Genes regulated, in both endothelial cell types, by coculture with both PC cell lines are represented. A significant threshold of a twofold change in gene expression corresponded to *p* < 0.001. Experiments were performed in duplicate. **D** Cytofluorimetric analyses of ITGAV (at the top), and of JAG1 (at the bottom) expression in HUVEC and HAEC untreated (CTRL) or treated with rhIL30 (50 ng/ml for 24 h and restimulation with 100 ng/ml for 3 h). Experiments were performed in duplicate. **E** Fold differences of mRNAs of angiogenesis-related genes between HUVEC cocultured with IL30-DU145 *versus* HUVEC cocultured with DU145 (dark green bars), or HUVEC cocultured with IL30-PC3 *versus* HUVEC cocultured with PC3 (light green bars). Only genes up- or down-regulated in both coculture conditions are represented. A significant threshold of a twofold change in gene expression corresponded to *p* < 0.001. Experiments were performed in duplicate. **F**, **G** Representative images from *n* = 3 experiments of a phospho-kinase array (array of 43 kinases in duplicate spots) performed on HUVEC (**F**) and HAEC (**G**), after treatment with rhIL30 (50 ng/ml for 3 h and subsequent restimulation with 100 ng/ml for 15 min). Phosphorylation of signaling molecules (on the left side of the panels) is represented by paired spots, according to intensity of phosphorylation. Histograms (on the right side of the panels) represent the amounts of phosphorylated proteins, expressed as mean pixel density, measured by ImageJ software, of each pair of spots present on the stained membrane. *p < 0.05, Student's t-test compared with CTRL. **H** Venn diagram representing the signaling proteins, which are phosphorylated in HUVEC (light blue circle) and HAEC (pink circle) after treatment with rhIL30. Overlapping circles illustrate only the proteins phosphorylated after rhIL30 treatment in both EC types

To assess the role of tumor membrane-anchored IL30 in PC-endothelium crosstalk, we next investigated angiogenesis-related gene expression in ECs after coculture with IL30-overexpressing PC cells. Contact with IL30-DU145 strongly upregulated the expression of *ITGAV, IGF1, TGFA, JAG1, CXCL1, CXCL10, HGF* and *EDN1*, whereas it downregulated the expression of *COL4A3* [30], in both HUVEC and HAEC (Fig. 3A). A wider range of genes, most of which endowed with angiogenic, angiocrine and inflammatory function, were upregulated in both HUVEC and HAEC following their coculture with IL30-PC3, such as *TGFB2* [31], *IGF1* [32], *JAG1* [33, 34], *CCL11/Eotaxin* [35], *NOS3* [36], *FGF2* [37] and *ENG/endoglin* [38] (Fig. 3B).

The upregulation of *CXCL10, EDN1, IGF1*, along with that of *ITGAV* and *JAG1* [39, 40], which was confirmed by flow cytometry, was shared by HUVEC and HAEC, after coculture with both IL30-DU145 and IL30-PC3 cells (Fig. 3C, D).

Intriguingly, analysis of the transcriptional profile of HUVEC, after contact with IL30 overexpressing DU145 or PC3 cells, revealed that, among the pro-angiogenic factors induced by this interaction, ANG [41] was the most upregulated (650 times in ECs cocultured with IL30-DU145, and 35 times in ECs cocultured with IL30-PC3 cells, whereas *ITGAV* upregulation ranked second, rising up to 594 times in ECs cocultured with IL30-DU145, and up to 24 times in ECs cocultured with IL30-PC3 cells) (Fig. 3E).

Since cytokine signaling pathways are commonly mediated through the phosphorylation of signal-dependent transcription factors, we carried out a phospho-kinase protein array using lysates of rhIL30-treated and untreated ECs (Fig. 3F, G). Seven proteins in HUVEC, including SRC, YES, GSK3α/β, and ten proteins in HAEC, including STAT3, STAT6, RSK 1/2, C-JUN, AKT, WNT and GSK3β, showed significant differences in phosphorylation after rhIL30 treatment. Most notably CREB [42, 43], GSK3β [44], HSP60 [45] and p53 [46], were significantly more phosphorylated in both HUVEC and HAEC (Fig. 3H, Supplementary Fig. S5), which can lead to endothelial dysfunction and regulate endothelial release of growth and inflammatory factors, as revealed by PCR array.

**Fig. 4:**
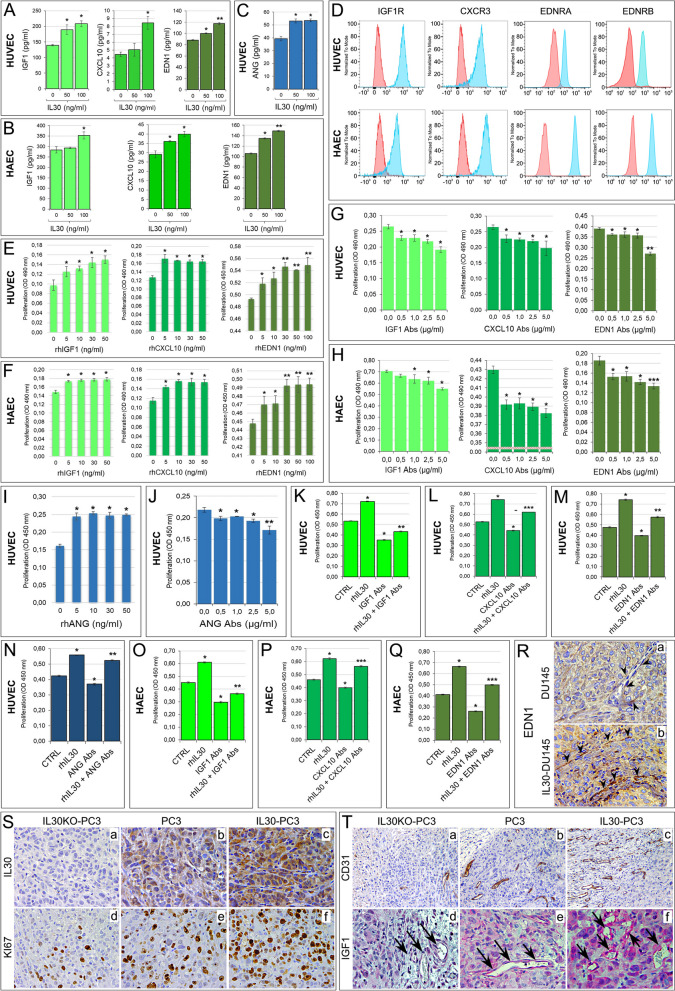
Upregulation of IGF1, CXCL10, EDN1 and ANG contributes to IL30-driven endothelial cell proliferation. **A**, **B** Elisa assays of IGF1, CXCL10 and EDN1 release by untreated (0 ng/ml) or rhIL30 treated (50-100 ng/ml) HUVEC (**A**) and HAEC (**B**). ANOVA: *p* < 0.01. *p < 0.01, Tukey HSD Test compared with untreated cells. **p < 0.01, Tukey HSD Test compared with cells treated with 50 ng/ml or untreated. Results are expressed as mean ± SD. **C** Elisa assay of ANG by untreated (0 ng/ml) or rhIL30 treated (50-100 ng/ml) HUVEC. ANOVA: *p* < 0.0001. *p < 0.01, Tukey HSD test compared with untreated cells. Results are expressed as mean ± SD. **D** Cytofluorimetric analyses of IGF1R, CXCR3, EDNRA and EDNRB expression in HUVEC (top) and HAEC (bottom). Red lines: isotype control. Experiments were performed in triplicate. **E**, **F** MTT assay of HUVEC (**E**) and HAEC (**F**), untreated (CTRL) or treated with rhIGF1, rhCXCL10 (5–50 ng/ml) or rhEDN1 (5–100 ng/ml). ANOVA: *p* < 0.001. *p < 0.05, Tukey HSD Test compared with 0 ng/ml. **p < 0.05, Tukey HSD Test compared with 0 and 5 ng/ml. Experiments were performed in triplicate and results are expressed as mean ± SD. **G**, **H** MTT assay of HUVEC (**G**) and HAEC (**H**), untreated (CTRL) or treated with anti-IGF1, anti-CXCL10 or anti-EDN1 Abs (0.5–5 μg/ml—48 h). ANOVA: *p* < 0.001. *p < 0.01, Tukey HSD Test compared with 0.0 µg/ml. **p < 0.05, Tukey HSD Test compared with 0.0, 0.5, 1.0 and 2.5 µg/ml. ***p < 0.05, Tukey HSD Test compared with 0.0, 0.5 and 1.0 µg/ml. Experiments were performed in triplicate and results are expressed as mean ± SD. **I**, **J** MTT assay of HUVEC untreated or treated with rhANG (5–50 ng/ml) (**I**) or with anti-ANG Abs (0.5–5 μg/ml) (**J**). ANOVA: *p* < 0.0001. *p < 0.05, Tukey HSD Test compared with 0 ng/ml or 0.0 µg/ml. **p < 0.01, Tukey HSD Test compared with 0, 0.5, 1.0 and 2.5 µg/ml. Experiments were performed in triplicate and results are expressed as mean ± SD. **K**, **L** MTT assay of HUVEC untreated (CTRL) or treated with rhIL30 (50 ng/ml), anti-IGF1 (**K**) or anti-CXCL10 (**L**) Abs (5 µg/ml), and rhIL30 + anti-IGF1 (**K**) or rhIL30 + anti-CXCL10 (**L**) Abs. ANOVA: *p* < 0.0001. *p < 0.01, Tukey HSD Test compared with CTRL. **p < 0.01, Tukey HSD Test compared with CTRL and cells treated with anti-IGF1 Abs. ***p < 0.01, Tukey HSD Test compared with CTRL and cells treated with rhIL30. Experiments were performed in triplicate and results are expressed as mean ± SD. **M**, **N** MTT assay of HUVEC untreated (CTRL) or treated with rhIL30 (50 ng/ml), anti-EDN1 (**M**) or anti-ANG (**N**) Abs (5 µg/ml), and rhIL30 + anti-EDN1 (**M**) or rhIL30 + anti-ANG (**N**) Abs. ANOVA: *p* < 0.0001. *p < 0.01, Tukey HSD Test compared with CTRL. **p < 0.01, Tukey HSD Test compared with CTRL and cells treated with rhIL30. Experiments were performed in triplicate and results are expressed as mean ± SD. **O**, **P**, **Q** MTT assay of HAEC untreated (CTRL) or treated with rhIL30 (50 ng/ml), anti-IGF1 (**O**) or anti-CXCL10 (**P**) or anti-EDN1 (**Q**) Abs (5 µg/ml), and rhIL30 + anti-IGF1 (**O**) or rhIL30 + anti-CXCL10 (**P**) or rhIL30 + anti-EDN1 (**Q**) Abs. ANOVA: *p* < 0.0001. *p < 0.01, Tukey HSD Test compared with CTRL. **p < 0.01, Tukey HSD Test compared with CTRL and cells treated with anti-IGF1 Abs. ***p < 0.01, Tukey HSD Test compared with CTRL and cells treated with rhIL30. Experiments were performed in triplicate and results are expressed as mean ± SD. **R** Immunohistochemistry shows that endothelin-1 (EDN1) expression is scanty in wild type DU145 tumors (a) and its endothelial network (arrowheads), while it is distinct in IL30 overexpressing DU145 tumors (b) and especially marked in the walls of its vascular network (arrowheads). Results from EV-tumors were comparable to wild type tumors. Magnification: X400. **S, T** Immunohistochemical features of IL30 knockout and overexpressing PC3 tumors *versus* wild type tumors (**S**, a-c) showing a different proliferation (**S**, d-f), vascularization (**T**, a-c) and expression of IGF1 (**T**, d-f, vessels indicated by arrows). IGF1 expression in the vascular endothelium was moderate in the wild type tumor, almost absent in the IL30KO tumor and increased in the IL30 overexpressing tumor, as indicated by the arrows. Results from EV-transfected and NTgRNA-tumors were comparable to wild type tumors. Magnification: X400; Ta-c, X200

To assess whether IL30 directly affects the production of these factors, both EC types were treated with different concentrations of rhIL30. ELISA assay revealed that both HUVEC and HAEC constitutively released IGF1 (139.67 ± 2.31 and 283.67 ± 15.28 pg/ml, respectively), CXCL10 (4.46 ± 0.29 and 29.08 ± 1.84 pg/ml, respectively), EDN1 (88.20 ± 0.90 and 106.00 ± 1.30 pg/ml, respectively), and that treatment with rhIL30 (50–100 ng/ml) significantly increased their production and release (Fig. 4A, B). In addition, treatment of HUVEC with rhIL30 substantially increased the basal release of ANG (Fig. 4C), therefore confirming the ability of tumor membrane-anchored IL30 to regulate the endothelial production of angiogenesis factors. Flow cytometry showed that both HUVEC and HAEC expressed IGF1R, CXCR3 (all three isoforms A, B and alt were expressed, as shown in the Supplementary Fig. S6), EDNRA and EDNRB (Fig. 4D), and treatment with recombinant IGF1, CXCL10, and EDN1 significantly increased (ANOVA: *p* < 0.001) their proliferation (Fig. 4E, F), which was inhibited (ANOVA: *p* < 0.001) by the treatment with neutralizing anti-IGF1, anti-CXCL10, or anti-EDN1 Abs (Fig. 4G, H). The treatment of HUVEC with recombinant ANG, a potent inducer of new blood vessel formation that binds to high-affinity, yet to be identified, endothelial cell-surface receptors [41], increased their proliferation, which was suppressed by anti-ANG Abs (Fig. 4I, J).

Notably, IL30-induced endothelial hyperproliferation was substantially reduced (ANOVA: *p* < 0.0001) by neutralizing antibodies against each of the angiogenesis stimulating factors (IGF1, CXCL10, EDN1, ANG), which also inhibited spontaneous EC proliferation, due to their baseline production and release (ANOVA: *p* < 0.0001) (Fig. 4K-Q). IGF1, CXCL10, EDN1 and ANG cooperate substantially in mediating IL30-driven endothelial hyperproliferation and are most likely involved in IL30 driven in vivo angiogenesis, since IL30 overexpressing tumors developed after xenograft implantation of IL30-PC3 or IL30-DU145 cells, which have demonstrated overgrowth and metastatic behavior [15], showed a prosperous vascularity, hyperproliferation and strong expression of these angiogenesis regulators when compared to control tumors, as reported in ref 15 and confirmed by data on PC3 tumor xenografts (Supplementary Table S5, Fig. 4R, S, T).

### Contact with Interleukin-30 knockout PC cells inhibits endothelial cell expression of angiogenesis regulatory genes, such as IGF1, CXCL6, TGFA and THBS2

To answer the question of whether abrogation of IL30 expression in PC cells could affect the transcriptional profile of contiguous ECs, we performed angiogenesis PCR array in ECs cocultured with IL30KO-DU145 or IL30KO-PC3 cells.

**Fig. 5:**
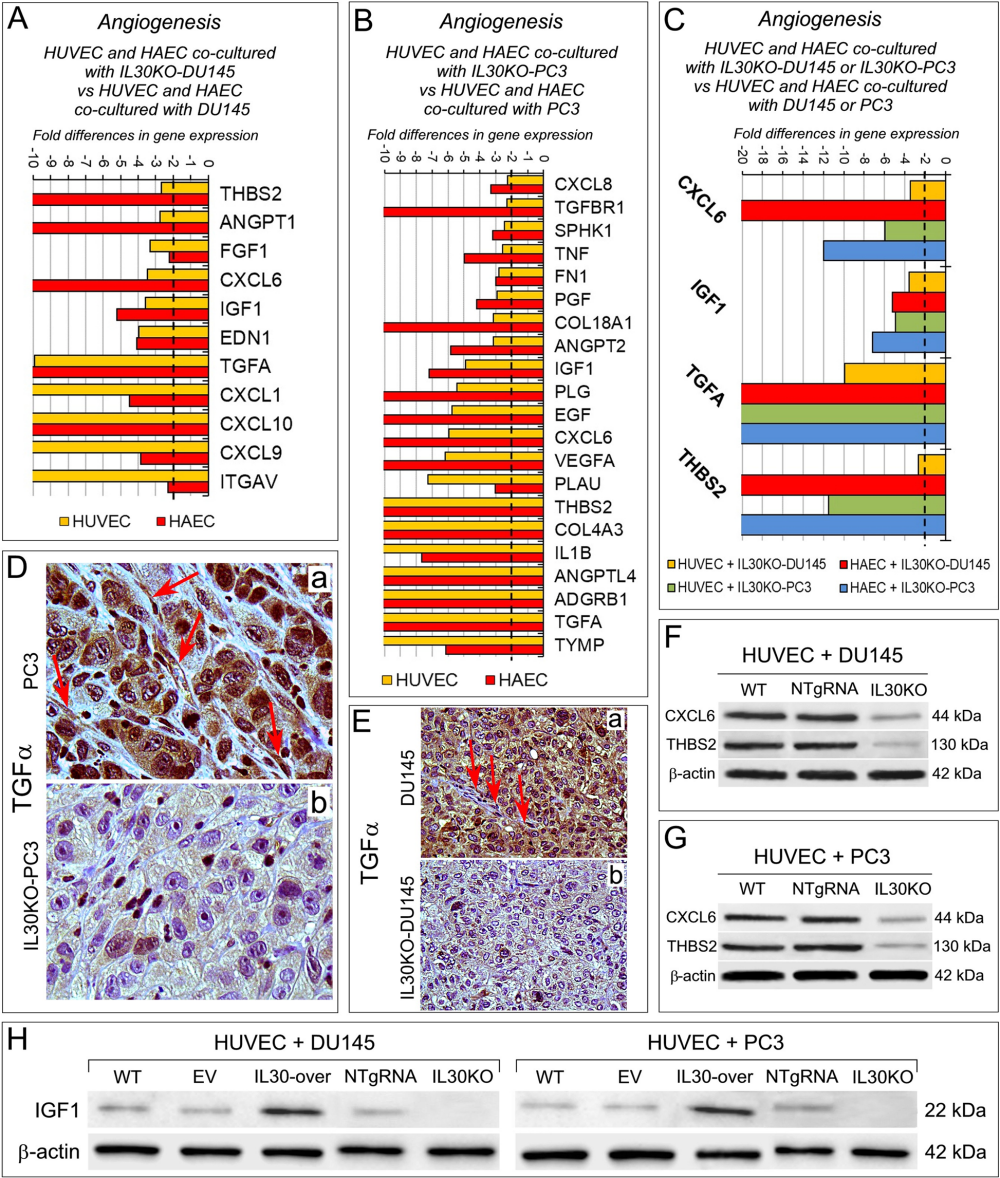
Contact with Interleukin-30 knockout PC cells downregulates angiogenesis-related genes in endothelial cells. **A** Fold differences of mRNAs of angiogenesis-related genes between HUVEC (yellow bars) or HAEC (red bars) cocultured with IL30KO-DU145 *versus* HUVEC, or HAEC, cocultured with wild type DU145. Results obtained from control NTgRNA-treated cells were comparable to those from wild type cells. A significant threshold of a twofold change in gene expression corresponded to *p* < 0.001. Experiments were performed in duplicate. **B** Fold differences of mRNAs of angiogenesis-related genes between HUVEC (yellow bars) or HAEC (red bars) cocultured with IL30KO-PC3 *versus* HUVEC, or HAEC, cocultured with wild type PC3. Results obtained from control NTgRNA-treated cells were comparable to those from wild type cells. A significant threshold of a twofold change in gene expression corresponded to *p* < 0.001. Experiments were performed in duplicate. **C** Fold differences of mRNAs of angiogenesis-related genes between HUVEC cocultured with IL30KO-DU145 (yellow bars), or IL30KO-PC3 (green bars), *versus* HUVEC cocultured with wild type DU145, or PC3, respectively. Fold differences of mRNAs of angiogenesis-related genes of HAEC cocultured with IL30KO-DU145 (red bars), or IL30KO-PC3 (blue bars) *versus* HAEC cocultured with wild type DU145, or PC3, respectively. Only genes that were regulated in both endothelial cell types by contact with both PC cell type are represented. A significant threshold of a twofold change in gene expression corresponded to *p* < 0.001. Experiments were performed in duplicate. **D**, **E** Immunohistochemistry showing that expression of TGFα was marked in wild type PC3 (**D**, a) and DU145 (**E**, a) and their vascular endothelial network (red arrows), whereas it was scanty in IL30KO-PC3 (**D**, b) and IL30KO-DU145 tumors (**E**, b) and almost absent in their vascular network. Results from NTgRNA-tumors were comparable to wild type tumors. Magnification: X400. **F**, **G** Western blot analysis of CXCL6 and THBS2 protein expression in ECs cocultured with IL30KO-DU145 (**F**) or IL30KO-PC3 (**G**) *versus* ECs cocultured with control (NTgRNA-treated) or wild type (WT) DU145 (**F**) or PC3 (**G**) cells. Representative images of experiments in triplicate. **H** Western blot analysis of IGF1 protein expression in ECs cocultured with IL30 overexpressing or knockout DU145 or PC3 cells. Representative images of experiments in triplicate

Compared to coculture with wild type DU145 cells, coculture with IL30KO-DU145 cells drastically inhibited, in both HUVEC and HAEC, the expression of a wide range of proangiogenic genes (Fig. 5A), including *IGF1, EDN1, CXCL10* and *ITGAV*, which were found to be upregulated in ECs cocultured with IL30 overexpressing PC cells (Fig. 3A, B, C).

Contact with IL30KO-PC3 cells led to the suppression of different proangiogenic genes, including *VEGFA, TGFA, ANGPT2* and *ANGPTL4* in both HUVEC and HAEC (Fig. 5B).

Only the inhibition of *CXCL6, IGF1, TGFA* and *THBS2* expression was shared by HUVEC and HAEC following their culture with IL30KO-DU145 or IL30KO-PC3 cells and confirmed at the protein level by WB or immunopathological examination of IL30 deficient tumors induced by IL30 gene deleted PC cell implantation in NSG mice (Supplementary Table S6; Fig. 5D, E, F, G, H and Supplementary Fig. S7). These findings suggest that the effects, on the endothelial gene signature, of the interaction with IL30 knockout PC cells is tumor specific.

### The crosstalk with the endothelium affects PC cell expression of immunity-related and PC driver genes, which is regulated by the overexpression of membrane-anchored IL30 in PC cells

The finding that contacts with PC cells and, even more so, their contact with IL30 overexpressing or deficient PC cells, could regulate the angiogenic profile of ECs, led us to wonder whether the endothelial-cancer cell crosstalk could also have impact on the immune profile and oncogenic potential of PC cells. To answer this question, the expression of immunoregulatory and PC driver genes was investigated by PCR array (Supplementary Tables S7 and S8) in wild type and IL30-overexpressing DU145 and PC3 cells cocultured with normal ECs.

**Fig. 6:**
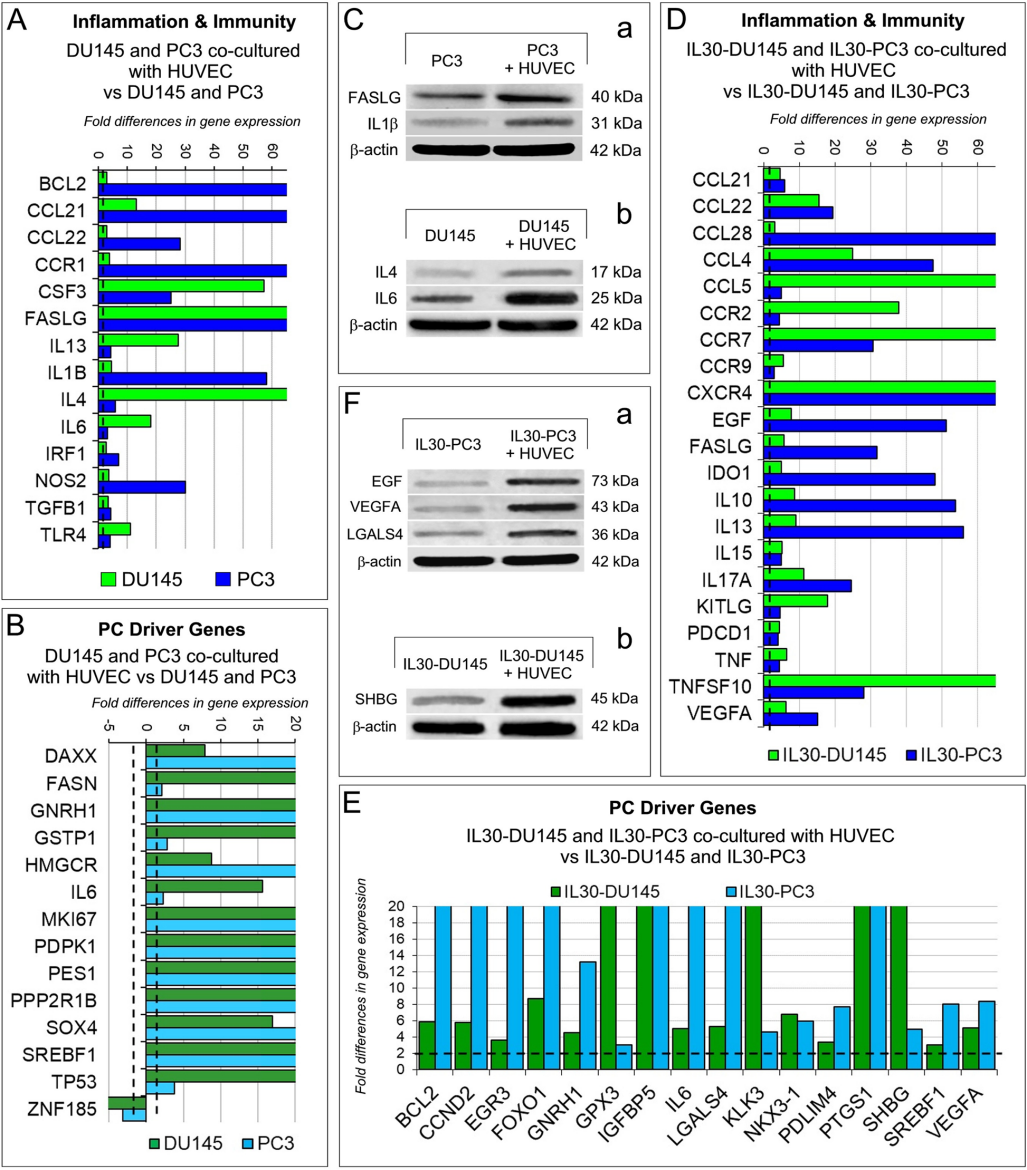
Regulation of inflammation and immunity genes, and prostate cancer driver genes in PC cells cocultured with endothelial cells. **A**, **B** Fold differences of mRNAs of *“inflammation & immunity”* genes (**A**) and of *“prostate cancer driver”* genes (**B**) between DU145, or PC3, cocultured with HUVEC *versus* DU145, or PC3, respectively. A significant threshold of a twofold change in gene expression corresponded to *p* < 0.001. Experiments were performed in duplicate. **C** Western blot analysis of FASLG, IL1β, IL4 and IL6 protein expression in PC3 (a) and DU145 (b) which were cultured with or without HUVEC. Representative images of three experiments. **D**, **E** Fold differences of mRNAs of *“inflammation & immunity”* genes (**D**) and of *“prostate cancer driver”* genes (**E**) between IL30-DU145 or IL30-PC3 cocultured with HUVEC *versus* DU145 or PC3, respectively. Results obtained from control EV-transfected cells were comparable to those from wild type cells. A significant threshold of a twofold change in gene expression corresponded to *p* < 0.001. Experiments were performed in duplicate. **F** Western blot analysis of EGF, VEGF, LGALS4 and SHBG protein expression in IL30-PC3 (a) and IL30-DU145 (b) which were cultured with or without HUVEC. Representative images of three experiments

Coculture with HUVEC determined, in both DU145 and PC3 cells, an impressive upregulation of proinflammatory and immunoregulatory genes, especially *BCL2, CCL21, CCL22, CCR1, CSF3, FASL, IL1B, IL4* and *NOS2* (Fig. 6A, C) and a consistent upregulation of prostate cancer driver genes*,* that included *DAXX, FASN, HMGCR, IL6, MKI67, PDPK1, PES1, SOX4* and *SREFB1* (Fig. 6B). Among prostate cancer driver genes, the tumor suppressor gene *ZNF185* was downregulated, whereas *GNRH1, PPP2R1B* and *TP53* were upregulated in both DU145 and PC3 cocultured with HUVEC *versus* cancer cells cultured alone. Furthermore, when overexpressing IL30, DU145 and PC3 cells showed, after coculture with ECs, a higher and broader expression of both inflammation and PC driver genes compared to monocultured IL30-DU145 or IL30-PC3 cells (Fig. 6D, E, F). The crosstalk with ECs, that had been stimulated by IL30 overexpression in PC cells, also shaped the transcriptional profile of PC cells by expanding the range and level of expression of immunity genes, such as those coding for chemokines, as *CCL28, CCL4* and *CCL5*; chemokine receptors, as *CCR2*, *CCR7, CCR9* and *CXCR4*; cytokines and growth factors, as *IL10, IL13, IL17A, EGF* and *VEGFA* (Fig. 6D). Similarly, after their culture with ECs, IL30-overexpressing PCs showed a dramatic upregulation of a wide range of oncogenes, which included *CCND2, EGR3, IGFBP5, KLK3, PDLIM4, PTGS1* and *SHBG* and a few tumor suppressors, such as *GPX3, FOXO1, MAX* and *NKX3-1* (Fig. 6E, F).

### Contact with the endothelium fosters the expression of immunoregulatory genes IL12B, NOS2, TNFA and CXCR5, and prostate cancer drivers SHBG, LGALS4 and GNRH1 in IL30 overexpressing tumor xenografts and in clinical PC samples

**Fig. 7:**
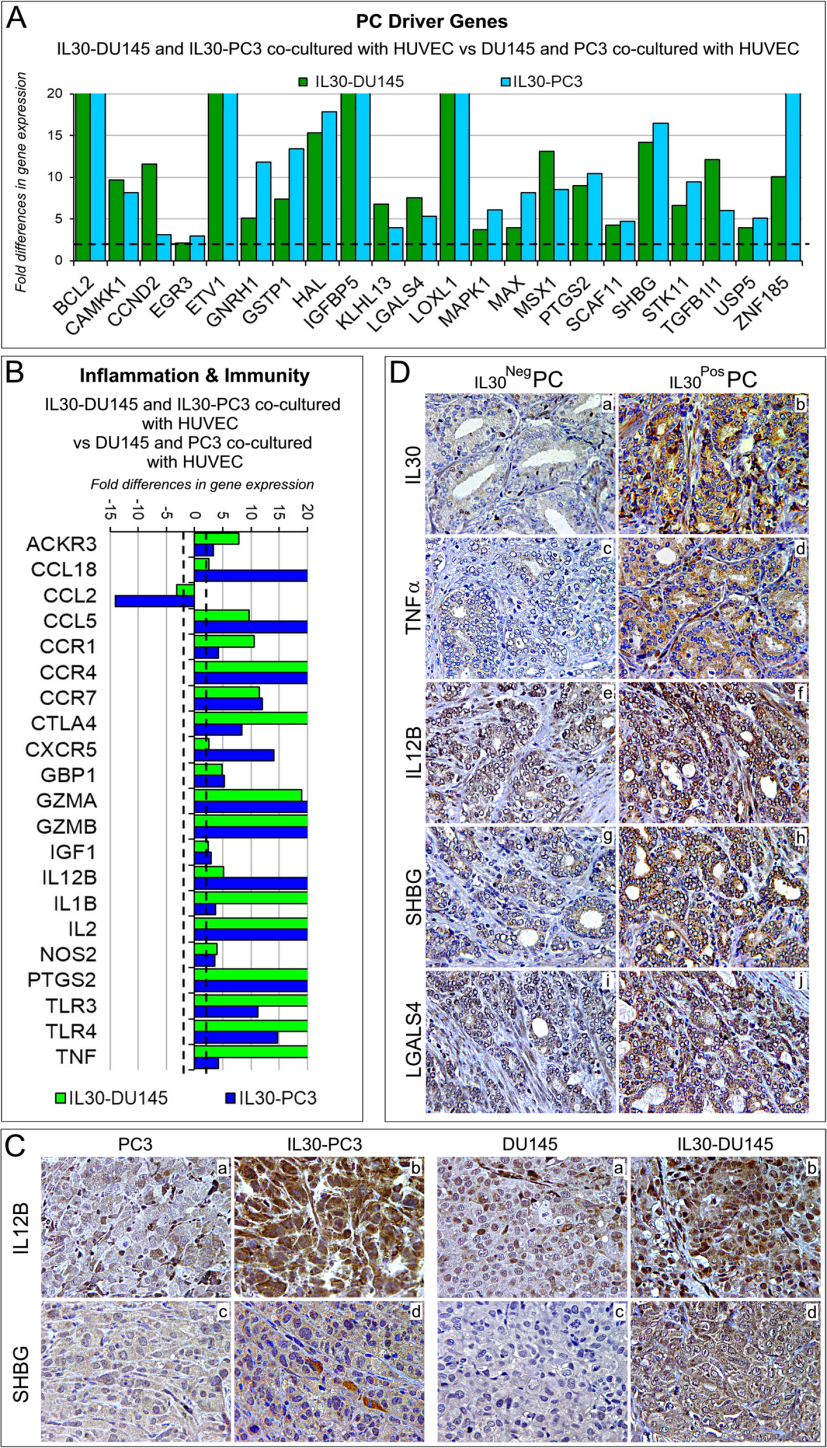
Regulation of inflammation and immunity genes, and prostate cancer driver genes in IL30 overexpressing PC cells cocultured with endothelial cells and in IL30 overexpressing tumor xenografts and clinical PC samples. **A**, **B** Fold differences of mRNAs of *“prostate cancer driver”* genes (**A**) or *“inflammation & immunity”* genes (**B**) between IL30-DU145 or IL30-PC3 cells cocultured with HUVEC *versus* DU145 or PC3 cocultured with HUVEC, respectively. Results obtained from control EV-transfected cells were comparable to those from wild type cells. A significant threshold of a twofold change in gene expression corresponded to *p* < 0.001. Experiments were performed in duplicate. **C** Immunohistochemical features of IL30 overexpressing PC3 and DU145 tumors showing a stronger expression of immunoregulatory genes (IL12B; a, b) and PC driver genes (SHBG; c, d) when compared to wild type tumors. Results from EV-transfected tumors were comparable to wild type tumors. Magnification: X400. **D** Expression of IL30 (a, b), TNFα (c, d), IL12B (e, f), SHBG (g, h) and LGALS4 (i, j) in PC tissues obtained from patients bearing IL30^Neg^ PC or IL30^Pos^ PC. Representative images of the immunohistochemical results are shown. Magnification: X400

Since in clinical PC samples cancer cells are in close contact with the endothelium, to assess whether the experimental findings have clinical relevance, we investigated in patient-derived PC samples the expression levels of IL30 along with that of immunoregulatory and PC driver genes, which were regulated in both IL30 overexpressing DU145, and PC3 cells after coculture with ECs when compared to wild type DU145, and PC3 cells, respectively, cocultured with ECs (Fig. 7A, B). Bioinformatic analysis was performed by using the *“Prostate Cancer Transcriptome Atlas”* (*PCTA*), the largest of the publicly available databases, which contains RNA-Seq data obtained from 1116 clinical PC specimens, with annotation of Gleason score, collected from 38 PC patient cohorts [47].

Among the PC driver genes that were found upregulated in IL30 overexpressing PC cells, after coculture with ECs (Fig. 7A), genes coding for *Galectin 4 (LGALS4), Gonadotropin Releasing Hormone 1 (GNRH1)*, and *Sex Hormone Binding Globulin (SHBG)* correlated with IL30 expression also in clinical PC samples, as assessed by bioinformatics of RNA-Seq data from the *PCTA* collection. Specifically, expression of *LGALS4* and *GNRH1* (*ρ* = 0.36 and *ρ* = 0.33, respectively; *p* < 0.01), and especially that of *SHBG* (*ρ* = 0.47; *p* < 0.01) positively correlated with IL30 expression.

Among the inflammation and immunity genes that were found to be upregulated in IL30 overexpressing PC cells, after coculture with ECs (Fig. 7B), genes coding for *Nitric Oxide Synthase 2 (NOS2)*, *Tumor necrosis Factor alpha (TNFA)*, *C-X-C chemokine receptor type 5 (CXCR5)* and *Interleukin-12B (IL12B)* correlated with IL30 expression also in clinical PC samples from the *PCTA* collection. Specifically, expression of *NOS2, TNFA, CXCR5* (*ρ* = 0.38, *ρ* = 0.37 and *ρ* = 0.36, respectively; *p* < 0.01), and primarily that of *IL12B* (*ρ* = 0.47; *p* < 0.01) positively correlated with IL30 expression. Moreover, immunohistochemical analysis confirmed, in vivo, overexpression of the aforementioned inflammation and cancer driver genes (*IL12B, SHBG, TNFA, LGALS4*) in IL30-overexpressing DU145 and PC3 tumors, compared to control tumors (Fig. 7C, Supplementary Fig. S8 and Supplementary Table S9).

Since IL12B (p40 subunit of the Interleukin 12 family of cytokines) has been found to protect PC cells from apoptosis [48], we next investigated the relationship between the expression of *IL12B* and that of apoptotic-related genes (Supplementary Table S2) in PC samples. The Spearman rank sum of RNA-Seq data from the *PCTA* database, demonstrated an inverse correlation between the transcriptional expression of *IL12B* and that of the genes involved in the apoptotic signaling pathway (*ρ* = -0.40; *p* < 0.01). Expression of apoptosis-related genes also showed an inverse correlation with the expression of *SHBG* (*ρ* = -0.33; *p* < 0.01) in PC sample database. The inverse correlation was stronger (*ρ* = -0.44; *p* < 0.01) when the combined expression levels of *IL12B* and *SHBG* were considered, suggesting that inhibition of apoptosis may be a further downstream mechanism that mediates the tumor promoting activity of IL30.

To assess at the protein level the correlations between gene expressions resulting from both experimental and bioinformatic findings, we next performed immunopathological analyses of tumor samples obtained from 80 patients (a cohort size which allowed the detection of a statistically significant correlation between two genes, with an 85% power and a 5% significance level) who underwent surgery for PC and were selected from our institutional Biobank by matching for Gleason score with PC patients of the *PCTA* collection (Table 1). Morphometric analysis confirmed, at protein level, the positive correlation between expression of IL30 and that of IL12B and SHBG (*ρ* = 0.50 and *ρ* = 0.52, respectively; *p* < 0.01) and, to a lesser extent, between IL30 positivity and immunostainings for LGALS4 (*ρ* = 0.32), GNRH1 (*ρ* = 0.31), NOS2 (*ρ* = 0.36), TNFA (*ρ* = 0.33) and CXCR5 (*ρ* = 0.33) (*p* < 0.01), therefore substantiating the biostatistical findings (Fig. 7D, Supplementary Fig. S9, the quantification of the immunohistochemical expression of inflammation and immunity genes, and prostate cancer driver genes, for each patient, is reported in the Supplementary Table S10) and highlighting the intricate network of immunoregulatory and cancer driver genes correlated with IL30 expression in the PC microenvironment.

## Discussion

The vascular bed of a tumor, including PC, supports its growth, is pivotal for its metastatic spread [49] and mediates communication with immune cells, thus playing a crucial role in the balance between tumor immune evasion and host anti-tumor immune response [50]. Here, we demonstrate that PC-EC contact-dependent signals promote endothelial proliferation, as revealed by Ki67 increase in ECs, and activation, as demonstrated by the endothelial expression of a variety of mediators, most of which have pleiotropic activities, primarily CXCL10 [18], CXCL9 [25], ANG [24], EDN1 [26, 27], TGFB2 [28] and TIMP3 [29], which may function as angiocrine factors, immunity or angiogenesis regulators. Although the range and level of expression of inflammation and angiogenesis-related genes strictly depends on the specific type of PC cell involved, contact with the PC cell generally shapes a pro-inflammatory and angiogenic endothelial gene signature.

Endothelial proliferation and inflammatory/angiogenesis gene expression induced by the contact with PC cells, are dramatically exacerbated by the juxtracrine signals released by cancer cells overexpressing membrane-bound IL30, which boosts endothelial expression of CXCL10, EDN1, IGF1, ANG, ITGAV and JAG1, promotes capillary sprouting and upregulates endothelial adhesion molecules, in particular P-selectin, which is essential to platelet and leukocyte adhesion and rolling [23] and is functional to the metastatic process [51, 52], and VCAM-1, which mediates monocytes and granulocytes adhesion and transmigration [23], and likely supports the monocyte/macrophage and granulocyte influx observed in IL30-overexpressing tumor xenograft [15].

Reprogramming of the endothelial transcriptional profile, due to contact with IL30-overexpressing PC cells, feeds significant autocrine growth loops, which are mediated by typical endothelial growth factors, such as IGF1 [32], ANG [39], and EDN1 [26], or by immunoregulatory molecules, such as CXCL10, which in addition to its role in leukocytes recruitment, has demonstrated to fuel cancer stem cell proliferation [18] and, depending on the dose and signaling pathway involved, to exert angiostatic or angiogenic effects [53, 54], as we observed in this context. Endothelial phenotype remodeling, by contact with IL30-overexpressing PC, is also demonstrated by the upregulation of ITGAV, which contributes to EC proliferation and migration [55] and promotes PC progression [56, 57], and the Notch ligand JAG1, which is essential for blood vessel formation and maturation [58] and is associated with metastasis and poor disease-free survival of PC patients [59, 60].

IL30 driven EC activation and inflammation is associated with the phosphorylation of a cascade of signaling proteins, which includes SRC, YES, STAT3, STAT6, RSK 1/2, C-JUN, AKT and, primarily CREB [42, 43], GSK-3α/β [44], HSP60 [45] and p53 [46] leading to endothelial dysfunction. CREB induces genes that regulate inflammation and vascular remodeling and maintains basal endothelial barrier function suppressing endothelial permeability due to thrombin, lipopolysaccharide, and VEGF [61]. GSK-3 negatively regulates endothelial cell migration and tube formation, therefore GSK-3β inhibition through phosphorylation can improve VEGF-driven angio-architecture and lumen formation during pathological angiogenesis [62, 63]. HSP60 is a multifaceted molecule with a wide range of cellular and tissue locations and functions, and its phosphorylation has been implicated in tumor immune evasion [64] and invasiveness [65] and delay of apoptosis activation [66]. The p53 tumor suppressor is implicated in endothelial dysfunction [67] and its phosphorylation contributes to the impairment of the endothelial antioxidant system [68].

Consistently with the amplification of the angiogenic circuitry driven by IL30 overexpression in PC cells, IL30 gene deletion, by CRISPR/Cas9-mediated genome editing, inhibits endothelial cell proliferation and turns off endothelial activation and inflammation, since a wide range of immunity and angiogenesis regulators, including EDN1, CXCL10, ITGAV, VEGFA, ANGPT2, ANGPT4, and primarily IGF1 [32], TGFA [69], CXCL6 [70] and THBS2 [71], were dramatically suppressed in ECs by contact with any of the IL30 gene-deleted PC cell type. The prevalent inhibition of pro-angiogenic over anti-angiogenic factors is consistent with the poor vascularity of IL30-defective and low metastatic tumors [15] and, by contrast, with the high microvessel density of IL30-overexpressing and rapidly progressing tumors ([16, 17], and data shown in this paper).

The crosstalk between endothelium and PC cells not only regulates the endothelial transcriptome, but inevitably impacts the immune profile and oncogenic program of tumor cells. Contact with the endothelium significantly upregulates PC cell expression of the anti-apoptotic gene, *BCL2*, growth factors, cytokines, chemokines and their receptors, especially *CSF3, IL1β, IL4, IL6, CCL21, CCL22, CCR1, NOS2*, which drives multiple oncogenic pathways [72] and *FASLG*, which may help to maintain tumor cells in a state of immune privilege by inducing apoptosis of anti-tumor immune effector cells [73]. Moreover, contact with ECs fosters PC cell expression of a wide range of oncogenes, such as *DAXX, FASN, GSTP1, MKI67, PDPK1, PES1, SOX4* and *SREBF1*, and a few tumor suppressors such as *GNRH1* [74], *PPP2R1B* [75] and *TP53* [76], whereas *ZNF185* [77] was downregulated.

Contact of IL30-overexpressing PC cells with the endothelium further increases their expression of growth factors and proinflammatory mediators, including *VEGFA, CCL28, CCL4, CCL5, CCR2, CCR7, CXCR4, IL10, IL13, IL17A* and promotes mechanisms of immune privilege by upregulating cancer cell expression of *FASLG, IDO1, KITLG* [78], apoptosis inducing ligand *TNFSF10/TRAIL *[79] and *PDCD1/PD-1*, which is involved in tumor initiation and progression [80].

Contact with the endothelium also fosters, in IL30-overexpressing cancer cells, a PC progression program as demonstrated by the dramatic upregulation of a wide range of PC driver genes, including *BCL2, CCND2, EGR3, GNRH1, IGFBP5, IL6, KLK3, PTGS1, SHBG, SREBF1* and *VEGFA*, that largely overwhelm the expression of tumor suppressors *FOXO1* [81], *GPX3* [82], NKX3-1 [83], and *PDLIM4* [84].

Bioinformatic analysis of gene expression profiles of PC samples from 1116 patients of the *PCTA collection* and immunohistochemistry of 80 PC samples from Gleason score-matched patients, emphasize the translational value of IL30 interference in the PC-endothelium crosstalk, highlighting a significant association between the expression of IL30 in clinical PC samples and that of immunoregulatory genes, such as *NOS2, TNFA, CXCR5* and, particularly, *IL12B* [48], and of prostate cancer driver genes, such as *LGALS4, GNRH1* and, particularly, *SHBG* [85, 86], which were found to be substantially upregulated when PC cells cocultured with EC cells overexpressed membrane-bound IL30. Finally, the strong inverse correlation determined by the Spearman rank sum of RNA-Seq data from the *PCTA* database, between the combined expression of *IL12B* and *SHBG* and the expression of apoptotic pathway genes, raises the possibility that the inhibition of programmed cell death may be a further pro-tumoral event downstream of the IL30-driven proinflammatory signaling cascade.

## Conclusions

A novel mechanism of IL30 regulation of tumor behavior has been demonstrated to be *via* the modulation of the cancer-endothelial cell crosstalk, which activates angiogenic, immunoregulatory and oncogenic pathways. Targeting IL30 may severely compromise the PC-EC relationship and counteract PC progression.

### Supplementary Information

**Additional file 1: Supplementary Fig. S1.** Cytofluorimetric analyses of endothelial cell marker expression in HUVEC (top of the panel), and HAEC (bottom of the panel). Both endothelial cell types expressed CD31/PECAM-1, CD34, CD54, CD309/VEGFR2 and vWF, but did not express CD45. Red lines: isotype control. Experiments were performed in triplicate. **Supplementary Fig. S2.** Western blot analysis showing that both HUVEC and HAEC express EBI3, but not the p28 (IL30) subunit of the heterodimeric (p28/EBI3) cytokine IL27. A representative image of triplicate experiments is shown. **Supplementary Fig. S3.** Cytofluorimetric analysis of cell apoptosis, by annexin V staining, in HUVEC (top of the panel) and HAEC (bottom of the panel) co-cultured or not with wild type DU145, IL30KO-DU145 or IL30-DU145. The negative annexin V staining indicates the absence of cells undergoing apoptosis in all conditions. Experiments were performed in triplicate. **Supplementary Fig. S4.** Western blot analysis of ANG, CXCL9, EDN1 and TGFB2 protein expression in HAEC co-cultured with DU145 (left side of the panel) or PC3 cells (right side of the panel). Representative images of three experiments. **Supplementary Fig. S5.** Western blot analysis of phosphorylated HSP60, p53, CREB and GSK3b proteins in ECs (HUVEC and HAEC) treated with rhIL30. **Supplementary Fig. S6.** Expression of CXCR3 isoforms in HAEC and HUVEC, as determined by RT-PCR. CXCR3A: 111 bp; CXCR3B: 79 bp; CXCR3-alt: 135 bp. **Supplementary Fig. S7.** Western blot analysis of CXCL6 and THBS2 protein expression in ECs (HAEC) co-cultured with IL30KO-DU145 (A) or IL30KO-PC3 (B) *versus* ECs co-cultured with control (NTgRNA-treated) or wild type (WT) DU145 or PC3 cells. Western blot analysis of IGF1 protein expression in ECs co-cultured with IL30 overexpressing or knockout DU145 or PC3 cells (C). Representative images of experiments in triplicate. **Supplementary Fig. S8.** Immunohistochemical analyses of PC3 (A) and DU145 (B) tumors show that expression of LGALS4 (a, b) and TNFa (c, d) is stronger in IL30 overexpressing tumors when compared to the respective wild type tumors (e, f). Results from EV-tumors were comparable to wild type tumors. Magnification: X400. **Supplemental Fig. S9.** Expression of CXCR5 in PC tissues obtained from patients bearing IL30^Neg^ PC or IL30^Pos^ PC. Inset shows a magnification of CXCR5 positive tumor cells. Representative images of the immunohistochemical study are shown. Magnification: X400. **Supplementary Table S1.** Antibodies used in flow cytometry. **Supplementary Table S2.** List of genes included in the apoptotic signaling pathway. **Supplementary Table S3.** Antibodies used in immunostaining. **Supplementary Table S4.** Gene list of the RT^2^ Profiler Human Angiogenesis PCR Array (#PAHS-024ZR). **Supplementary Table S5.** Immunohistochemical analysis of IL30 expression, microvessel density and proliferation index in wild type, IL30 gene transfected or deleted tumors and control EV-transfected or NTgRNA-treated tumors. **Supplementary Table S6.** Immunohistochemical analysis of inflammation and immunity genes in wild type and IL30 gene knockout tumors. **Supplementary Table S7.** Gene list of the RT^2^ Profiler Human Prostate Cancer PCR Array (#PAHS-135ZR). **Supplementary Table S8.** Gene list of the RT^2^ Profiler Human Cancer Inflammation & Immunity Crosstalk PCR Array (#PAHS-181Z). **Supplementary Table S9.** Immunohistochemical analysis of inflammation and immunity genes and prostate cancer driver genes in wild type and IL30 gene transfected tumors. **Supplementary Table S10.** Morphometric evaluation of IL30 and immunoregulatory and prostate cancer driver gene expression in prostate cancer samples from patients of the validation cohort*.

## Data Availability

The datasets used and/or analysed during the current study are available from the corresponding author on reasonable request. Expression data, from tumor samples of the “*Prostate Cancer Transcriptome Atlas”* (*PCTA*) collection, were derived from the following resource available in the public domain: the *PCTA* website (http://www.thepcta.org).

## Abbreviations

ANG: Angiogenin
BC: Breast cancer
Cas9: CRISPR associated protein 9
CTRL: Control
CRISPR: Clustered regularly interspaced short palindromic repeats
CXCL6: C-X-C motif chemokine ligand 6
CXCL9: C-X-C motif chemokine ligand 9
CXCL10: C-X-C motif chemokine ligand 10
CXCR5: C-X-C chemokine receptor type 5
EBI3: Epstein-Barr Virus Induced 3
EC: Endothelial cells
EDN1: Endothelin 1
EGF: Epidermal growth factor
EV: Empty Vector
GNRH1: Gonadotropin-releasing hormone 1
HAEC: Human Aortic endothelial cells
HUVEC: Human umbilical endothelial cells
IGF1: Insulin-like growth factor 1
IL: Interleukin
KO: Knockout
LV: Lentiviral-DNA
LGALS4: Galectin-4
LSM: Laser scanning microscope
MDSCs: Myeloid-derived suppressor cells
MVD: Microvessel density
NOS2: Nitric oxide synthase 2
NTgRNA: Non-targeting guide RNA-treated
PC: Prostate cancer
PCTA: Prostate Cancer Transcriptome Atlas
SHBG: Sex hormone-binding globulin
TGFB2: Transforming growth factor beta 2
THBS2: Thrombospondin 2
TNFA: Tumor necrosis factor alpha
VEGFA: Vascular endothelial growth factor A
