# Supplementary material for: Interleukin-30 subverts prostate cancer-endothelium crosstalk by fostering angiogenesis and activating immunoregulatory and oncogenic signaling pathways
Source: J Exp Clin Cancer Res. 2023 Dec 12;42:336. doi: 10.1186/s13046-023-02902-y (PMC10714661; doi:10.1186/s13046-023-02902-y)
Supplement: Supplementary file 1 — Additional file 1: Supplementary Fig. S1. Cytofluorimetric analyses of endothelial cell marker expression in HUVEC (top of the panel), and HAEC (bottom of the panel). Both endothelial cell types expressed CD31/PECAM-1, CD34, CD54, CD309/VEGFR2 and vWF, but did not express CD45. Red lines: isotype control. Experiments were performed in triplicate. Supplementary Fig. S2. Western blot analysis showing that both HUVEC and HAEC express EBI3, but not the p28 (IL30) subunit of the heterodimeric (p28/EBI3) cytokine IL27. A representative image of triplicate experiments is shown. Supplementary Fig. S3. Cytofluorimetric analysis of cell apoptosis, by annexin V staining, in HUVEC (top of the panel) and HAEC (bottom of the panel) co-cultured or not with wild type DU145, IL30KO-DU145 or IL30-DU145. The negative annexin V staining indicates the absence of cells undergoing apoptosis in all conditions. Experiments were performed in triplicate. Supplementary Fig. S4. Western blot analysis of ANG, CXCL9, EDN1 and TGFB2 protein expression in HAEC co-cultured with DU145 (left side of the panel) or PC3 cells (right side of the panel). Representative images of three experiments. Supplementary Fig. S5. Western blot analysis of phosphorylated HSP60, p53, CREB and GSK3b proteins in ECs (HUVEC and HAEC) treated with rhIL30. Supplementary Fig. S6. Expression of CXCR3 isoforms in HAEC and HUVEC, as determined by RT-PCR. CXCR3A: 111 bp; CXCR3B: 79 bp; CXCR3-alt: 135 bp. Supplementary Fig. S7. Western blot analysis of CXCL6 and THBS2 protein expression in ECs (HAEC) co-cultured with IL30KO-DU145 (A) or IL30KO-PC3 (B) versus ECs co-cultured with control (NTgRNA-treated) or wild type (WT) DU145 or PC3 cells. Western blot analysis of IGF1 protein expression in ECs co-cultured with IL30 overexpressing or knockout DU145 or PC3 cells (C). Representative images of experiments in triplicate. Supplementary Fig. S8. Immunohistochemical analyses of PC3 (A) and DU145 (B) tumors show that expression of LGALS [file 13046_2023_2902_MOESM1_ESM.docx]

**Supplementary Methods**

**PCR Array and Real-Time RT-PCR**

RNA extraction was performed by using the RNeasy Mini Kit (#74104; Qiagen, Hilden, Germany), and reverse-transcribed with the RT2 First Strand Kit (#330401; Qiagen). PCR array analyses were run on a Qiagen Rotor Gene Q (RRID:SCR_018976), using the RT² Profiler Human Angiogenesis PCR Array (#PAHS-024ZR), RT² Profiler Human Cancer Inflammation & Immunity Crosstalk PCR Array (#PAHS-181Z) and the RT² Profiler™ Human Prostate Cancer PCR Array (#PAHS-135Z) (all from Qiagen) (Supplementary Tables S1, 3 and 4). The results from each plate were normalized to the median value of a set of housekeeping genes. Changes in the gene expression were calculated using the ΔΔCt method. Results from experiments performed in triplicate were pooled and analyzed with the manufacturer’s software. A significant threshold of a 2-fold change in gene expression corresponded to a *p*<0.001.

For the analysis of IL30 and CXCR3 expression in human cells, the real-time RT-PCR reaction was performed with the Quantifast SYBR Green PCR Kit (#204054; Qiagen) and a MiniOpticon System (#CFB-3120; Bio-Rad, Hercules, CA, USA). The primers for human IL30 (Human_IL27p28_1_SG QuantiTect Primer Assay, #QT00236250) and HPRT (Human_HPRT1_1_SG QuantiTect Primer Assay, #QT00059066) were purchased from Qiagen, whereas the primers for the CXCR3 isoforms (CXCR3A forward 5′-ACCCAGCAGCCAGAGCACC-3′; CXCR3A reverse 5′-TCATAGGAAGAGCTGAAGTTCTCCA-3′; CXCR3B forward 5′-TGCCAGGCCTTTACACAGC-3′; CXCR3B reverse 5′-TCGGCGTCATTTAGCACTTG-3′; CXCR3-alt forward 5′-CCAATACAACTTCCCACAGGGGT-3′; CXCR3-alt reverse 5′-GTCTCAGACCAGGATGAATCCCG-3) were designed and synthesized by Sigma-Aldrich Corporation (St. Louis, MO, USA).

For the analysis of IL30 mRNA levels, melting curve analysis was done to assess the specificity of PCR product and the relative quantification of mRNA was done according to the comparative threshold cycle method, with HPRT as calibrator, using the Bio-Rad CFX Manager software.

For the identification of human CXCR3 isoforms, the PCR products were separated on a 2% agarose gel, stained with ethidium bromide, and DNA bands were visualized with a Transilluminator 2000 (#170-8110; Bio-Rad, Hercules, CA, USA).

**Western blot**

For total protein extraction, cells were collected by centrifugation and lysed with ice cold RIPA Lysis buffer (#89900; Thermo Fisher Scientific, Waltham, MA, USA), supplemented with Protease and Phosphatase Inhibitors Cocktail (#78446; Thermo Fisher Scientific, Waltham, MA, USA). Total proteins were then quantified using the Bradford assay. Subsequently, whole cell lysates were loaded on Mini-PROTEAN TGX Gels 4-20% (#4561094; Bio-Rad, Hercules, CA, USA) and proteins were transferred from the gels on Immuno-Blot PVDF Membranes (#1620177; Bio-Rad, Hercules, CA, USA) in transfer buffer (glycine, tris [pH 8.4] and methanol), using Mini Trans-Blot Cell apparatus (#1703989; Bio-Rad). Membranes containing the transferred proteins were then blocked with 5% milk (#M7409; Sigma-Aldrich, St. Louis, MO, USA) in TBST and, subsequently, probed with primary and horseradish peroxidase conjugated secondary antibodies, following standard procedures. The following primary and secondary antibodies were used: mouse anti-human ANG (R&D Systems, Cat# MAB265, RRID AB_2227142); mouse anti-human phospho-CREB (R and D Systems Cat# MAB6906, RRID:AB_10972977); mouse anti-human CXCL6 (R&D Systems Cat# MAB333, RRID:AB_2086869); rabbit anti-human CXCL9 (Thermo Fisher Scientific, Cat# 701117 RRID:AB_2532396); rat anti-human EBI3 (LSBio, LifeSpan, Cat# LS-B5764-50, RRID:AB_10914746); rabbit anti-human EdN1 (Thermo Fisher Scientific, Cat# PA5-85430 RRID:AB_2792570); rabbit anti-human EGF (Santa Cruz Biotechnology, Cat# sc-275, RRID:AB_631417); mouse anti-human FASLG (Thermo Fisher Scientific Cat# MA5-44029, RRID:AB_2912961); rabbit anti-human phospho-GSK3β (R and D Systems Cat# MAB25062, RRID:AB_10889646); mouse anti-human phospho-HSP60 (R and D Systems Cat# MAB1800, RRID:AB_2118930); rat anti-human IGF1 (R&D Systems Cat# MAB2912); rabbit anti-human IL1β (Abcam, Cat# ab216995, RRID:AB_2894877); mouse anti-human IL4 (Thermo Fisher Scientific, Cat# MIL4I, RRID:AB_417057); rabbit anti-human IL6 (Abcam, Cat# ab22938, RRID:AB_2861234); rabbit anti-human IL-27A (IL30) (Abcam, Cat# ab118910, RRID:AB_10898806); rabbit anti-human LGALS4 (Biorbyt, Cat# orb30395, RRID:AB_10922155); rabbit anti-human phospho-p53 (R and D Systems Cat# AF1043, RRID:AB_354559); mouse anti-human SHBG (R&D Systems, Cat# MAB2656, RRID:AB_2187875); mouse anti-human TGFB2 (Santa Cruz Biotechnology, Cat# sc-374659, RRID:AB_10988781); rabbit anti-human THBS2 (Thermo Fisher Scientific Cat# PA5-95532, RRID:AB_2807334); mouse anti-human VEGF-A (Abcam, Cat# ab1316, RRID:AB_299738); goat anti-rabbit IgG (H + L)-HRP Conjugate (Sigma-Aldrich, Cat# A0545, RRID:AB_257896); rabbit anti-rat IgG (whole molecule)−Peroxidase (Sigma-Aldrich Cat# A5795, RRID:AB_258259) and rabbit anti-mouse IgG (whole molecule)-Peroxidase (Sigma-Aldrich, Cat# A9044, RRID:AB_258431). β-actin (Sigma-Aldrich Cat# A2228, RRID: AB_476697) was used as loading control. Membranes were then washed with TBST and developed with Pierce ECL Western Blotting Substrate (#32106; Thermo Fisher Scientific, Waltham, MA, USA).

**Histology, Immunohistochemistry and Morphometric Analyses**

To eliminate inter-operator bias and to improve data reproducibility, assessment of proliferation index, microvessel density and expression of immunoregulatory genes and prostate cancer driver genes in tumor samples was performed, by light microscopy, at ×400 in an 85,431.59 μm^2^ field, on single immunostained tissue section, with a Leica Imaging Workstation and QWin image analysis software (Leica QWin, RRID:SCR_018940), which ensures the following highly reproducible steps: 1) image acquisition; 2) conversion of RGB image (true colors) to binary image (black and white); 3) filtering to remove noise; 4) counting of immunostained cells; 5) measurement of positively stained areas, combining the evaluation of both the widening and the strength of the staining. Six to eight high-power fields were analyzed for each section and three sections per sample were evaluated. The histopathological evaluation was performed excluding necrotic areas. Results were expressed as mean percentage ± SD of positive cells/number of total cells (Ki67) per field, or as mean percentage ± SD of positively stained areas/total area (expression of IL30, immunoregulatory and prostate cancer driver genes in tumor xenografts) of the examined fields at ×400 (85 431.59 μm^2^). Microvessels were identified as small tubes or circles marked by CD31 Abs and results were expressed as mean ± SD of positive vessels/field. Patients’ tumour samples were defined as IL30 negative (IL30^Neg^ PC) or IL30 positive (IL30^Pos^ PC), based on the parameters described in Sorrentino et al. (*J Hematol Oncol. 2022;15:145*)*,* and reprocessed with the computerized image analysis system. Expression values of IL30, immunoregulatory genes and prostate cancer driver genes, in tumor samples from PC patients of the validation cohort, were represented as the mean percentage of the positively stained area/total field area, after scanning six to eight high-power fields per section and three sections per sample.


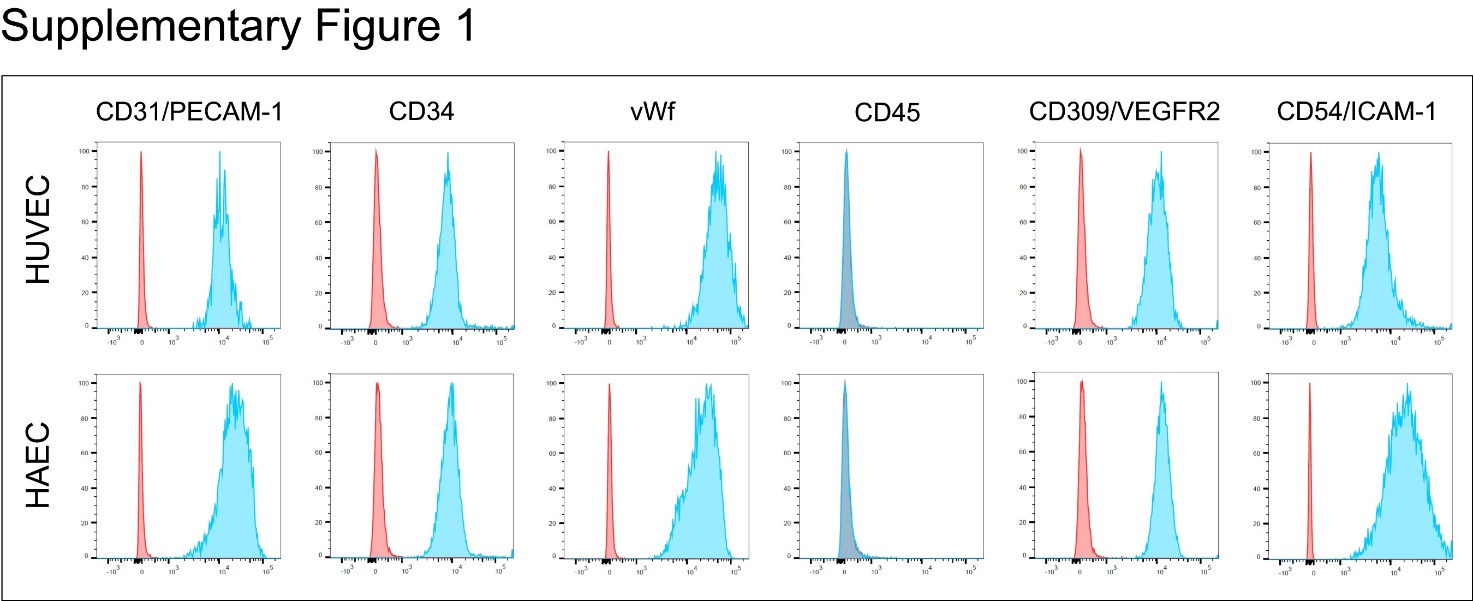


**Supplementary Fig. S1.** Cytofluorimetric analyses of endothelial cell marker expression in HUVEC (top of the panel), and HAEC (bottom of the panel). Both endothelial cell types expressed CD31/PECAM-1, CD34, vWF, CD309/VEGFR2 and CD54/ICAM-1, but did not express CD45. Red lines: isotype control. Experiments were performed in triplicate.


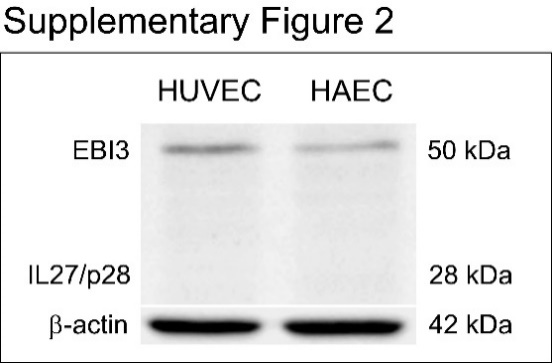


**Supplementary Fig. S2.** Western blot analysis showing that both HUVEC and HAEC express EBI3, but not the p28 (IL30) subunit of the heterodimeric (p28/EBI3) cytokine IL27. A representative image of triplicate experiments is shown.


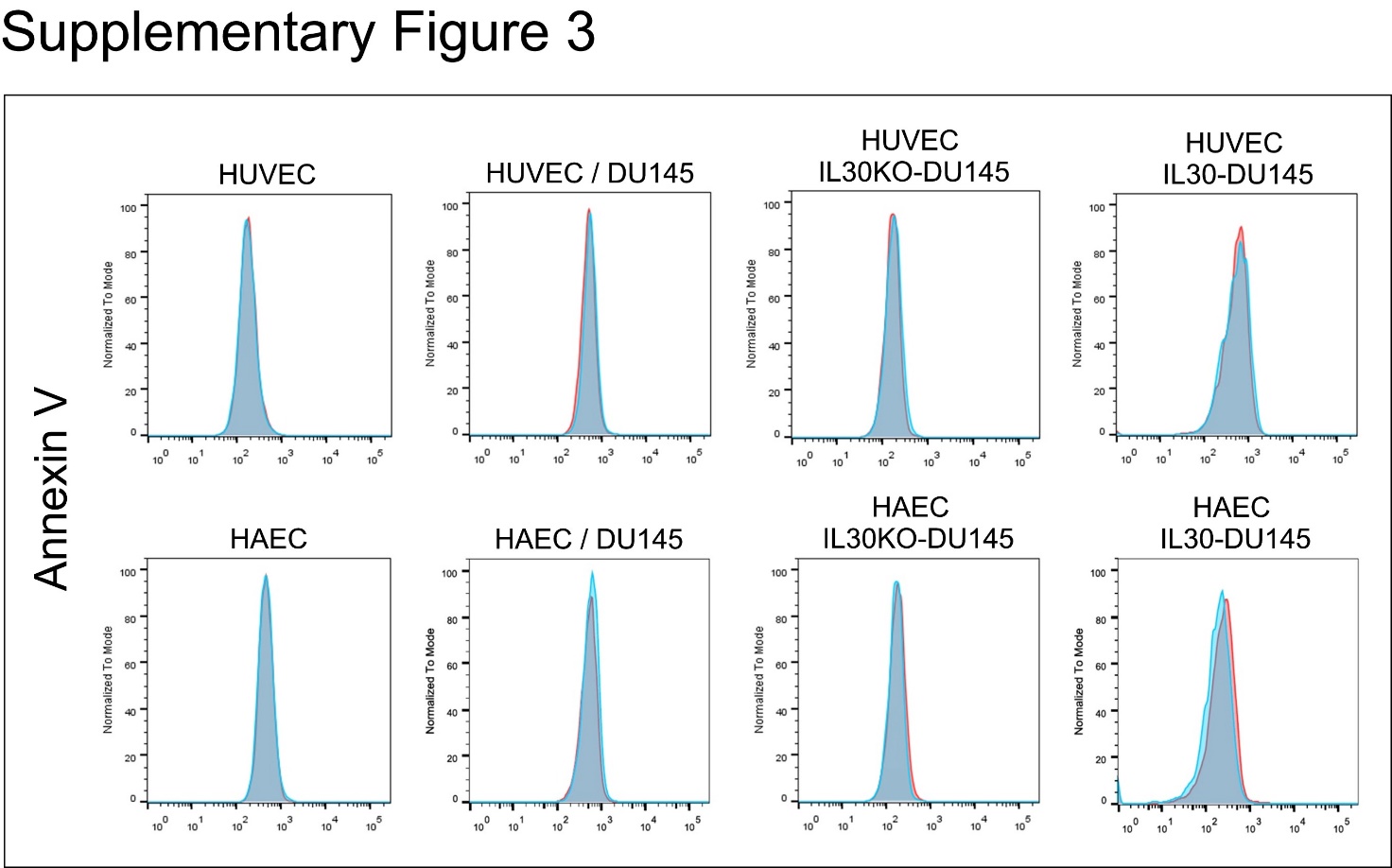


**Supplementary Fig. S3.** Cytofluorimetric analysis of cell apoptosis, by annexin V staining, in HUVEC (top of the panel) and HAEC (bottom of the panel) co-cultured or not with wild type DU145, IL30KO-DU145 or IL30-DU145. The negative annexin V staining indicates the absence of cells undergoing apoptosis in all conditions. Experiments were performed in triplicate.

**
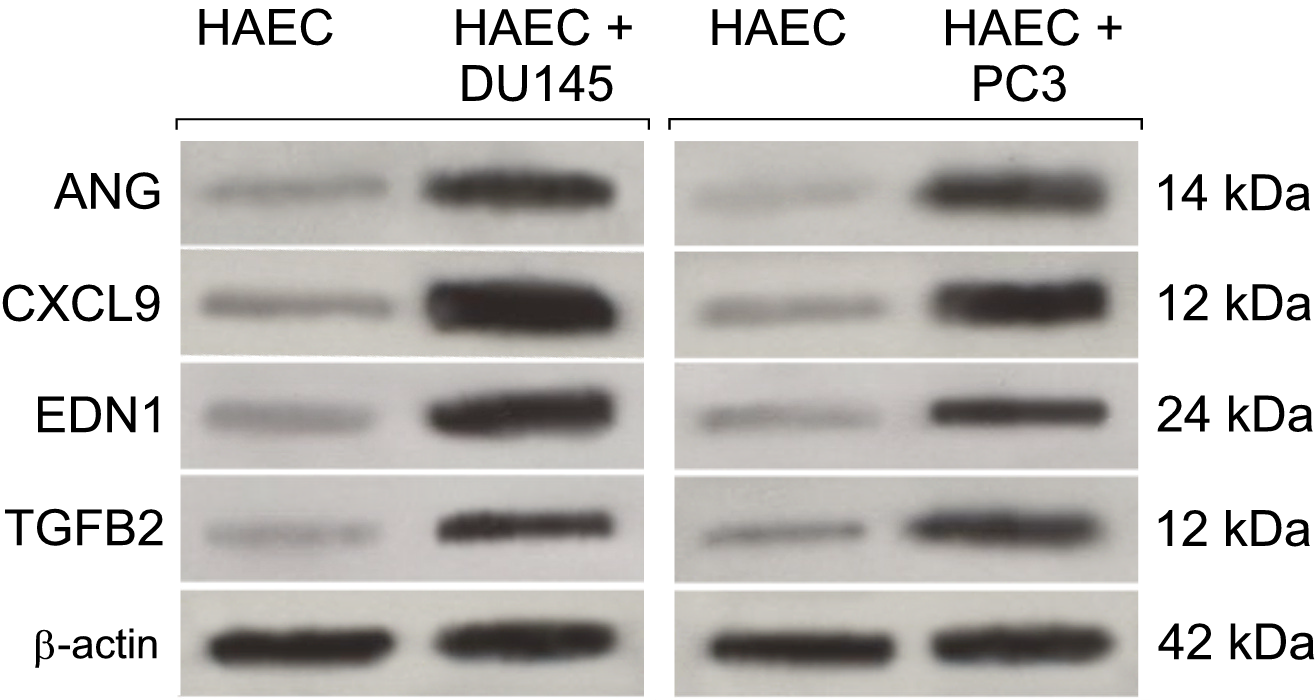
**

**Supplementary Fig. S4.** Western blot analysis of ANG, CXCL9, EDN1 and TGFB2 protein expression in HAEC co-cultured with DU145 (left side of the panel) or PC3 cells (right side of the panel). Representative images of three experiments.

**
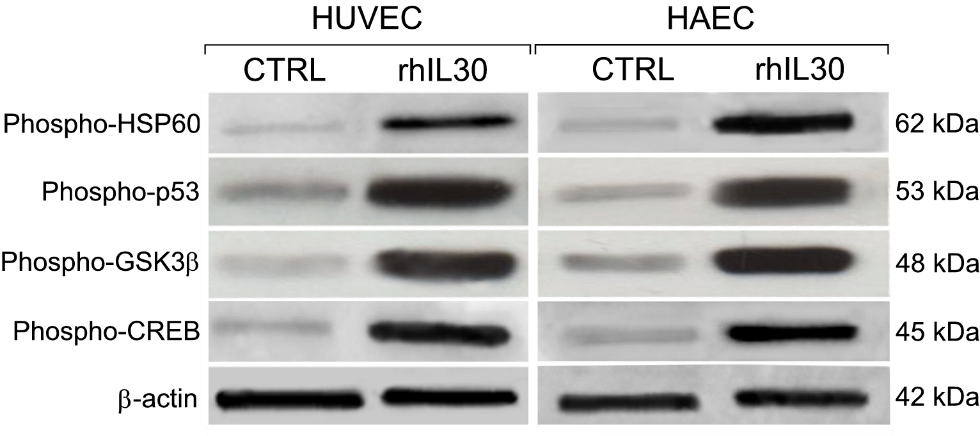
**

**Supplementary Fig. S5.** Western blot analysis of phosphorylated HSP60, p53, CREB and GSK3β proteins in ECs (HUVEC and HAEC) treated with rhIL30.


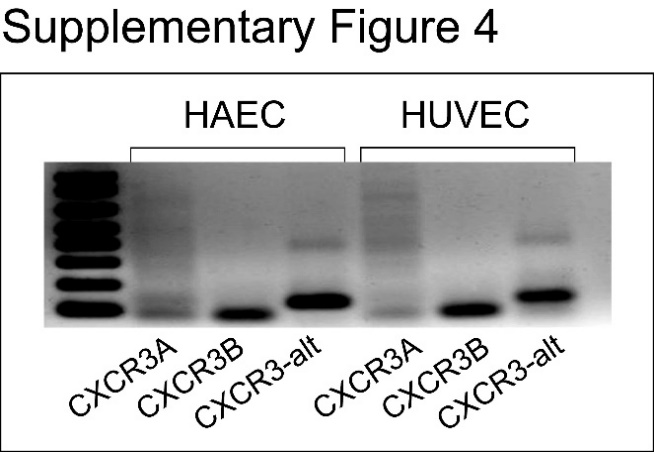


**Supplementary Fig. S6.** Expression of CXCR3 isoforms in HAEC and HUVEC, as determined by RT-PCR. CXCR3A: 111 bp; CXCR3B: 79 bp; CXCR3-alt: 135 bp.

**A**

**B**


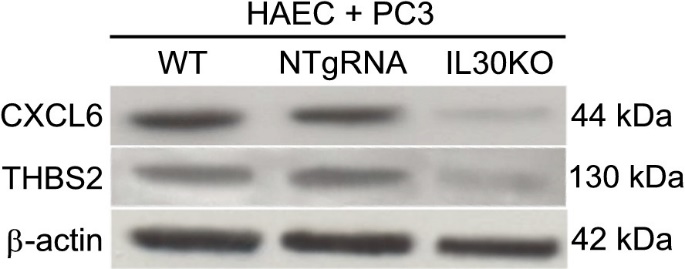

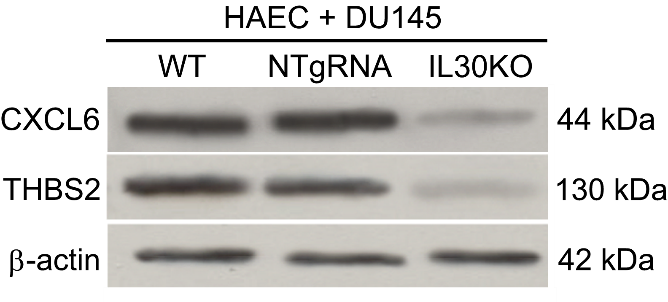


**
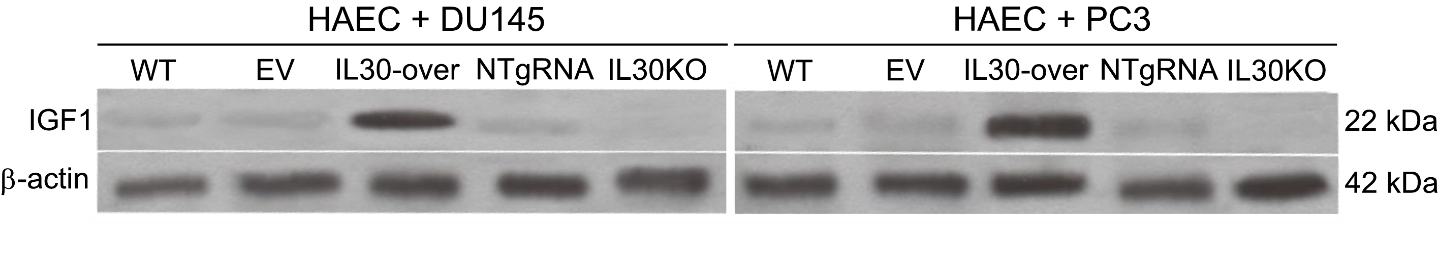
**

**C**

**Supplementary Fig. S7.** Western blot analysis of CXCL6 and THBS2 protein expression in ECs (HAEC) co-cultured with IL30KO-DU145 (**A**) or IL30KO-PC3 (**B**) versus ECs co-cultured with control (NTgRNA-treated) or wild type (WT) DU145 or PC3 cells. Western Blot analysis of IGF1 protein expression in ECs co-cultured with IL30 overexpressing or knockout DU145 or PC3 cells (**C**). Representative images of experiments in triplicate.


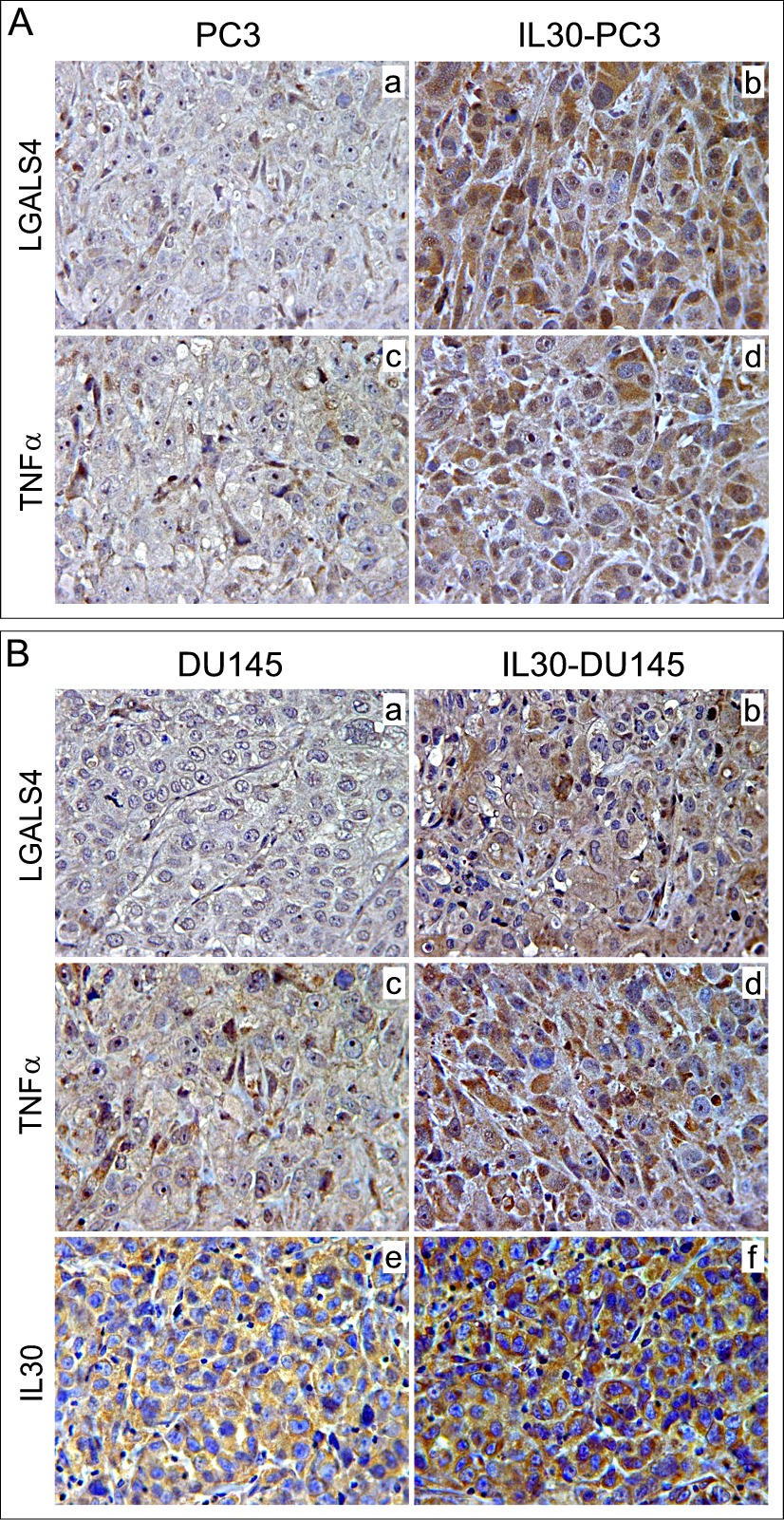


**Supplementary Fig. S8.** Immunohistochemical analyses of PC3 (**A**) and DU145 (**B**) tumors show that expression of LGALS4 (**a, b**) and TNFα (**c, d**) is stronger in IL30 overexpressing tumors when compared to the respective wild type tumors (**e, f**). Results from EV-tumors were comparable to wild type tumors. Magnification: X400.


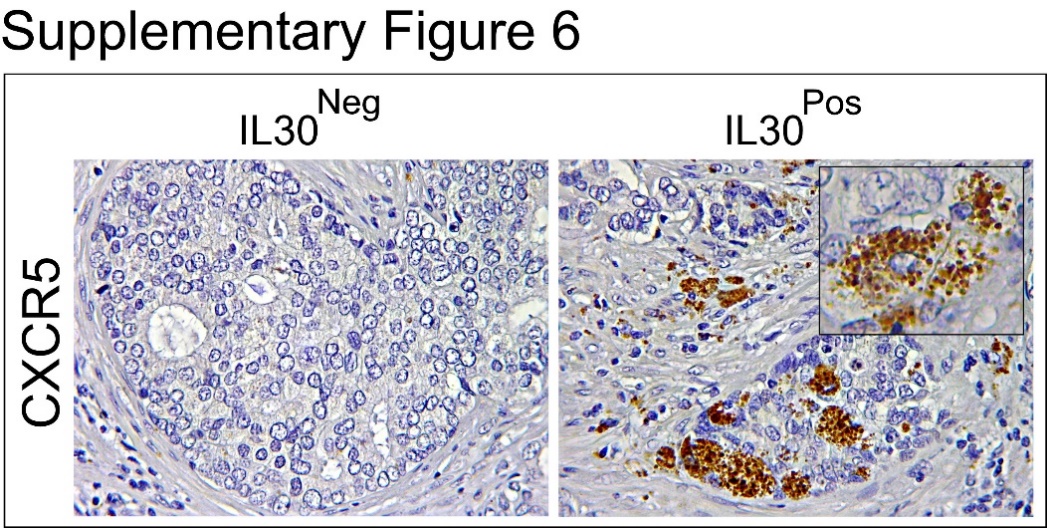


**Supplemental Fig. S9.** Expression of CXCR5 in PC tissues obtained from patients bearing IL30^Neg^ PC or IL30^Pos^ PC. Inset shows a magnification of CXCR5 positive tumor cells. Representative images of the immunohistochemical study are shown. Magnification: X400.

**Supplementary Table S1.** Antibodies used in flow cytometry

| **Antibody** | **Format** | **Research Resource Identifiers (RRIDs)** | **Source** |
| --- | --- | --- | --- |
|  |  |  |  |
| ***Anti-human*** |  |  |  |
| CD130 | FITC | RRID:AB_868803 | Abcam, Cambridge, UK |
| Annexin V | FITC | RRID:AB_2665412 | BD Biosciences, Franklin  Lakes, NJ, USA |
| CD126 | PE | RRID:AB_39427 | “ |
| CXCR3 | APC | RRID:AB_398481 | “ |
| EPCAM | PerCP-Cy5.5 | RRID:AB_400263 | “ |
| CD31 | FITC | RRID:AB_2733636 | Miltenyi Biotec, Bergisch  Gladbach, Germany |
| CD34 | PE | RRID:AB_2726281 | “ |
| CD45 | FITC | RRID:AB_265991 | “ |
| CD54  (ICAM-1) | PE | RRID:AB_2752194 | “ |
| CD62P  (P-selectin) | PE | RRID:AB_2733830 | “ |
| CD102  (ICAM-2) | PE | RRID:AB_2726616 | “ |
| CD106  (VCAM-1) | PE | RRID:AB_2801824 | “ |
| CD309  (VEGFR2) | PE | RRID:AB_2733728 | “ |
| EDNRA | Unconjugated | Cat: MAB65381 | R&D System, Minneapolis, MN, USA |
| EDNRB | Unconjugated | RRID:AB_10973827 | “ |
| ITGAV | Unconjugated | RRID:AB_2280706 | “ |
| JAG1 | Unconjugated | RRID:AB_35471 | “ |
| CD119 (IFNGR1) | PE | RRID:AB_11150056 | Thermo Fisher Scientific,  Waltham, MA, USA |
| FVIII | Unconjugated | RRID:AB_11001165 | “ |
| IGFR1 | APC | RRID:AB_1907365 | “ |
| IL30 | eFluor 660 | RRID:AB_11149127 | “ |
| Ki67 | APC | RRID:AB_2688057 | “ |
|  |  |  |  |

**Supplementary Table S2.** List of genes included in the apoptotic signaling pathway

| **Symbol** | **Gene Name** |
| --- | --- |
|  |  |
| *ADD1* | adducin 1 |
| *AIFM3* | apoptosis inducing factor mitochondria associated 3 |
| *ANKH* | ANKH inorganic pyrophosphate transport regulator |
| *ANXA1* | annexin A1 |
| *APP* | amyloid beta precursor protein |
| *ATF3* | activating transcription factor 3 |
| *AVPR1A* | arginine vasopressin receptor 1A |
| *BAX* | BCL2 associated X, apoptosis regulator |
| *BCAP31* | B cell receptor associated protein 31 |
| *BCL10* | BCL10 immune signaling adaptor |
| *BCL2L1* | BCL2 like 1 |
| *BCL2L10* | BCL2 like 10 |
| *BCL2L11* | BCL2 like 11 |
| *BCL2L2* | BCL2 like 2 |
| *BGN* | biglycan |
| *BID* | BH3 interacting domain death agonist |
| *BIK* | BCL2 interacting killer |
| *BIRC3* | baculoviral IAP repeat containing 3 |
| *BMF* | Bcl2 modifying factor |
| *BMP2* | bone morphogenetic protein 2 |
| *BNIP3L* | BCL2 interacting protein 3 like |
| *BRCA1* | BRCA1 DNA repair associated |
| *BTG2* | BTG anti-proliferation factor 2 |
| *BTG3* | BTG anti-proliferation factor 3 |
| *CASP1* | caspase 1 |
| *CASP2* | caspase 2 |
| *CASP3* | caspase 3 |
| *CASP4* | caspase 4 |
| *CASP6* | caspase 6 |
| *CASP7* | caspase 7 |
| *CASP8* | caspase 8 |
| *CASP9* | caspase 9 |
| *CAV1* | caveolin 1 |
| *CCNA1* | cyclin A1 |
| *CCND1* | cyclin D1 |
| *CCND2* | cyclin D2 |
| *CD14* | CD14 molecule |
| *CD2* | CD2 molecule |
| *CD38* | CD38 molecule |
| *CD44* | CD44 molecule |
| *CD69* | CD69 molecule |
| *CDC25B* | cell division cycle 25B |
| *CDK2* | cyclin dependent kinase 2 |
| *CDKN1A* | cyclin dependent kinase inhibitor 1A |
| *CDKN1B* | cyclin dependent kinase inhibitor 1B |
| *CFLAR* | CASP8 and FADD like apoptosis regulator |
| *CLU* | clusterin |
| *CREBBP* | CREB binding protein |
| *CTH* | cystathionine gamma-lyase |
| *CTNNB1* | catenin beta 1 |
| *CYLD* | CYLD lysine 63 deubiquitinase |
| *DAP* | death associated protein |
| *DAP3* | death associated protein 3 |
| *DCN* | decorin |
| *DDIT3* | DNA damage inducible transcript 3 |
| *DFFA* | DNA fragmentation factor subunit alpha |
| *DIABLO* | diablo IAP-binding mitochondrial protein |
| *DNAJA1* | DnaJ heat shock protein family member A1 |
| *DNAJC3* | DnaJ heat shock protein family member C3 |
| *DNM1L* | dynamin 1 like |
| *DPYD* | dihydropyrimidine dehydrogenase |
| *EBP* | EBP cholestenol delta-isomerase |
| *EGR3* | early growth response 3 |
| *EMP1* | epithelial membrane protein 1 |
| *ENO2* | enolase 2 |
| *ERBB2* | erb-b2 receptor tyrosine kinase 2 |
| *ERBB3* | erb-b2 receptor tyrosine kinase 3 |
| *EREG* | epiregulin |
| *ETF1* | eukaryotic translation termination factor 1 |
| *F2* | coagulation factor II, thrombin |
| *F2R* | coagulation factor II thrombin receptor |
| *FAS* | Fas cell surface death receptor |
| *FASLG* | Fas ligand |
| *FDXR* | ferredoxin reductase |
| *FEZ1* | fasciculation and elongation protein zeta 1 |
| *GADD45A* | growth arrest and DNA damage inducible alpha |
| *GADD45B* | growth arrest and DNA damage inducible beta |
| *GCH1* | GTP cyclohydrolase 1 |
| *GNA15* | G protein subunit alpha 15 |
| *GPX1* | glutathione peroxidase 1 |
| *GPX3* | glutathione peroxidase 3 |
| *GPX4* | glutathione peroxidase 4 |
| *GSN* | gelsolin |
| *GSR* | glutathione-disulfide reductase |
| *GSTM1* | glutathione S-transferase mu 1 |
| *GUCY2D* | guanylate cyclase 2D, retinal |
| *H1-0* | H1.0 linker histone |
| *HGF* | hepatocyte growth factor |
| *HMGB2* | high mobility group box 2 |
| *HMOX1* | heme oxygenase 1 |
| *HSPB1* | heat shock protein family B member 1 |
| *IER3* | immediate early response 3 |
| *IFITM3* | interferon induced transmembrane protein 3 |
| *IFNB1* | interferon beta 1 |
| *IFNGR1* | interferon gamma receptor 1 |
| *IGF2R* | insulin like growth factor 2 receptor |
| *IGFBP6* | insulin like growth factor binding protein 6 |
| *IL18* | interleukin 18 |
| *IL1A* | interleukin 1 alpha |
| *IL1B* | interleukin 1 beta |
| *IL6* | interleukin 6 |
| *IRF1* | interferon regulatory factor 1 |
| *ISG20* | interferon stimulated exonuclease gene 20 |
| *JUN* | Jun proto-oncogene, AP-1 transcription factor subunit |
| *KRT18* | keratin 18 |
| *LEF1* | lymphoid enhancer binding factor 1 |
| *LGALS3* | galectin 3 |
| *LMNA* | lamin A/C |
| *LUM* | lumican |
| *MADD* | MAP kinase activating death domain |
| *MCL1* | MCL1 apoptosis regulator, BCL2 family member |
| *MGMT* | O-6-methylguanine-DNA methyltransferase |
| *MMP2* | matrix metallopeptidase 2 |
| *NEDD9* | neural precursor cell expressed, developmentally down-regulated 9 |
| *NEFH* | neurofilament heavy chain |
| *PAK1* | p21 activated kinase 1 |
| *PDCD4* | programmed cell death 4 |
| *PDGFRB* | platelet derived growth factor receptor beta |
| *PEA15* | proliferation and apoptosis adaptor protein 15 |
| *PLAT* | plasminogen activator, tissue type |
| *PLCB2* | phospholipase C beta 2 |
| *PLPPR4* | phospholipid phosphatase related 4 |
| *PMAIP1* | phorbol-12-myristate-13-acetate-induced protein 1 |
| *PPP2R5B* | protein phosphatase 2 regulatory subunit B'beta |
| *PPP3R1* | protein phosphatase 3 regulatory subunit B, alpha |
| *PPT1* | palmitoyl-protein thioesterase 1 |
| *PRF1* | perforin 1 |
| *PSEN1* | presenilin 1 |
| *PSEN2* | presenilin 2 |
| *PTK2* | protein tyrosine kinase 2 |
| *RARA* | retinoic acid receptor alpha |
| *RELA* | RELA proto-oncogene, NF-kB subunit |
| *RETSAT* | retinol saturase |
| *RHOB* | ras homolog family member B |
| *RHOT2* | ras homolog family member T2 |
| *RNASEL* | ribonuclease L |
| *ROCK1* | Rho associated coiled-coil containing protein kinase 1 |
| *SAT1* | spermidine/spermine N1-acetyltransferase 1 |
| *SATB1* | SATB homeobox 1 |
| *SC5D* | sterol-C5-desaturase |
| *SLC20A1* | solute carrier family 20 member 1 |
| *SMAD7* | SMAD family member 7 |
| *SOD1* | superoxide dismutase 1 |
| *SOD2* | superoxide dismutase 2 |
| *SPTAN1* | spectrin alpha, non-erythrocytic 1 |
| *SQSTM1* | sequestosome 1 |
| *TAP1* | transporter 1, ATP binding cassette subfamily B member |
| *TGFB2* | transforming growth factor beta 2 |
| *TGFBR3* | transforming growth factor beta receptor 3 |
| *TIMP1* | TIMP metallopeptidase inhibitor 1 |
| *TIMP2* | TIMP metallopeptidase inhibitor 2 |
| *TIMP3* | TIMP metallopeptidase inhibitor 3 |
| *TNF* | tumor necrosis factor |
| *TNFRSF12A* | TNF receptor superfamily member 12A |
| *TNFSF10* | TNF superfamily member 10 |
| *TOP2A* | DNA topoisomerase II alpha |
| *TSPO* | translocator protein |
| *TXNIP* | thioredoxin interacting protein |
| *VDAC2* | voltage dependent anion channel 2 |
| *WEE1* | WEE1 G2 checkpoint kinase |
| *XIAP* | X-linked inhibitor of apoptosis |
|  |  |

**Supplementary Table S3.** Antibodies used in immunostaining

| **Antibody** | **Clone** | **Origin** | **Research Resource Identifiers (RRIDs)** | **Source** |
| --- | --- | --- | --- | --- |
|  |  |  |  |  |
| CD31* | JC/70A | Mouse | RRID:AB_307284 | Abcam, Cambridge, UK |
| CXCR5 |  | Rabbit | RRID:AB_444728 | “ |
| IGF1 |  | Rabbit | RRID:AB_308724 | “ |
| IL30 |  | Rabbit | RRID:AB_10898806 | “ |
| TNFα | 52B83 | Mouse | RRID:AB_302615 | “ |
| Ki67 | MIB1 | Mouse | RRID:AB_2142367 | Agilent, Santa Clara, CA, USA |
| LGALS4 |  | Rabbit | RRID:AB_2885342 | GeneTex, Hsinchu City, Taiwan |
| IL12β |  | Rabbit | #LS‑C804118 | LSBio, Seattle, WA, USA |
| EpCAM* | REA764 | Human | RRID:AB_2904949 | Miltenyi Biotec, Bergisch Gladbach, Germany |
| SHBG | 980027 | Mouse | #MAB26563 | R&D Systems,  Minneapolis, MN, USA |
| NOS2 |  | Rabbit | RRID:AB_2152867 | Santa Cruz Biotechnology, Dallas, TX, USA |
| TGFα |  | Rabbit | RRID:AB_2853550 | Thermo Fisher Scientific, Waltham, MA, USA |
|  |  |  |  |  |

*Antibodies used in immunofluorescent stainings.

**Supplementary Table S4.** Gene list of the RT² Profiler Human Angiogenesis PCR Array (#PAHS-024ZR)

| **Gene categories** | **Gene symbols** |
| --- | --- |
|  |  |
| **Angiogenic Growth Factors** | ***Growth Factors & Receptors*:** ANG, ANGPT1, ANGPT2, ANPEP, FGF1, FGF2 (BFGF), FLT1 (VEGFR1), JAG1, KDR (VEGFR3), NRP1, NRP2, PGF, TYMP, VEGFA, VEGFB, VEGFC, VEGFD (VEGFD).  ***Cell Adhesion Molecules*:** ADGRB1 (BAI1), COL4A3, CXCL8 (IL8), NRP1, NRP2.  ***Extracellular Matrix (ECM) Molecules:*** ANGPTL4, F3, PECAM1, PF4, PROK2, SERPINE1 (PAI-1), SERPINF1.  ***Other Angiogenic Factors*:** HIF1A, NOS3 (eNOS), SPHK1 |
|  |  |
| **Other Angiogenic Factors** | ***Cytokines*:** CCL11 (Eotaxin), CCL2 (MCP-1), CXCL1 (GRO1, GROa, SCYB1), CXCL10 (INP10), CXCL5 (ENA-78, LIX), CXCL6 (GCP-2), CXCL9 (MIG), EDN1, IFNA1, IFNG, IL1B, IL6, MDK, TNF.  ***Growth Factors & Receptors:*** CCN2, EFNA1, EFNB2, EGF, EPHB4, FGFR3, HGF, IGF1, ITGB3, PDGFA, S1PR1, TEK (TIE-2, TIE2), TGFA, TGFB1, TGFB2, TGFBR1 (ALK5).  ***Cell Adhesion Molecules:*** CCL11 (Eotaxin), CCL2 (MCP-1), CCN2, CDH5, COL18A1, ENG (EVI-1), ERBB2 (HER-2, NEU) ,FN1, ITGAV, ITGB3, S1PR1, THBS1 (TSP-1), THBS2.  ***Extracellular Matrix (ECM) Molecules:*** CNMD, LEP (Leptin), MMP14, MMP2, MMP9, PLAU (UPA), PLG, TIMP1, TIMP2, TIMP3*.*  ***Other Angiogenic Factors:*** AKT1, HPSE, ID1, NOTCH4, PTGS1 (COX1), TIE1*.* |
|  |  |

**Supplementary Table S5.** Immunohistochemical analysis of IL30 expression, microvessel density and proliferation index in wild type, IL30 gene transfected or deleted tumors and control EV-transfected or NTgRNA-treated tumors

|  | **WT-PC3** | **EV-PC3** | **IL30-PC3** | **NTgRNA-PC3** | **IL30KO-PC3** | **ANOVA**  ***p* value^§^** |
| --- | --- | --- | --- | --- | --- | --- |
| **IL30*** | 6.0 ± 0.5 | 5.9 ± 0.8 | 18.0 ± 5.5^‡^ | 6.2 ± 0.9 | 0.4 ± 0.3^#^ | <0.0001 |
| **Proliferation**  **Index (%)*** | 51.73±8.78 | 50.96±7.74 | 70.31±7.63^‡^ | 48.06±4.24 | 33.74±6.90^#^ | <0.0001 |
| **MVD*** | 11.74±3.22 | 11.98±3.81 | 22.95±5.38^‡^ | 12.49±3.34 | 4.39±1.56^#^ | <0.0001 |

*Microvessel density, proliferation index and IL30 positivity were assessed by light microscopy, at ×400 in an 85431.59 μm^2^ field, on single stained sections, with Qwin image analysis software (version 2.7), as described in the Supplementary Methods. Results are expressed as mean ± SD.

^§^ One-way ANOVA for comparisons between all groups.

^‡^ *p*<0.01, Tukey's HSD test compared with WT-PC3 and EV-PC3.

^#^ *p*<0.01, Tukey's HSD test compared with WT-PC3, EV-PC3 and NTgRNA-PC3.

**Supplementary Table S6.** Immunohistochemical analysis of inflammation and immunity genes in wild type and IL30 gene knockout tumors

| **Inflammation & Immunity Genes** | **Human Tumor Xenografts** | | | |
| --- | --- | --- | --- | --- |
|  | **WT-PC3*** | **IL30KO-PC3** | **WT-DU145*** | **IL30KO-DU145** |
|  |  | | | |
| **TGFα^§^** | 18.2 ± 5.3 | 3.4 ± 0.5^‡^ | 19.0 ± 6.6 | 4.8 ± 0.7^‡^ |
|  | | | | |

* Results from control Non-targeting guide RNA (NTgRNA) treated tumors were comparable to results from wild type (WT) tumors.

^§^ TGFα expression values are represented as the mean percentage ± SD of positively stained areas/total area of the examined fields (85431.59 μm^2^) at ×400.

^‡^ Values significantly different (Student's *t*-test: *p*<0.01) from corresponding values in WT-PC3 and WT-DU145 tumors.

**Supplementary Table S7.** Gene list of the RT² Profiler Human Prostate Cancer PCR Array (#PAHS-135ZR)

| **Gene categories** | **Gene symbols** |
| --- | --- |
|  |  |
| **Differentially Methylated Promoters** | APC, AR, CAV1, CCNA1, CDH1 (E-Cadherin), CDKN2A (P16INK4A), DKK3, DLC1, EDNRB, GPX3, GSTP1, MGMT (AGT), MSX1, PDLIM4 (RIL), PTGS2 (COX2), RARB, RASSF1, SFRP1, SLC5A8, TIMP2, TNFRSF10D, ZNF185. |
|  |  |
| **Up-Regulated in Prostate Cancer** | ARNTL (BMAL1), CAMSAP1, DDX11, ECT2, ETV1, HAL, IGFBP5, KLK3, MTO1, PDPK1, RBM39, SOCS3, SOX4, SUPT7L. |
|  |  |
| **Down-Regulated in Prostate Cancer** | CCND2, CLN3, GCA, IGF1, LGALS4, LOXL1, PPP2R1B, SFRP1, SLC5A8, TFPI2, USP5. |
|  |  |
| **Metastatic Potential** | CREB1, KLHL13, MAX, NDRG3, PES1, SCAF11, SEPT7. |
|  |  |
| **Androgen Receptor Signaling** | AR, CAV1, CCND1, DAXX, EGFR (ERBB1), FOXO1, GNRH1, IGF1, IL6, NFKB1, NRIP1, PTEN, SHBG, TGFB1I1, TIMP2, TIMP3, VEGFA. |
|  |  |
| **AKT & PI3 Kinase Signaling** | AKT1, AR, BCL2, CCND1, CCND2, CDH1 (E-Cadherin), CDKN2A (P16INK4A), EGFR (ERBB1), FOXO1, GNRH1, IGF1, IL6, MAPK1 (ERK2), NFKB1, PDPK1, PTEN, TIMP2, TIMP3, TNFRSF10D, TP53 (p53), VEGFA. |
|  |  |
| **PTEN Signaling** | AKT1, EGFR (ERBB1), GNRH1, IGF1, IL6, MAPK1 (ERK2), PDPK1, PTEN, TIMP2, TIMP3, TP53 (p53), VEGFA. |
|  |  |
| **Apoptosis** | BCL2, CASP3, CDKN2A (P16INK4A), EGFR (ERBB1), ETV1, GNRH1, IGF1, IL6, MAPK1 (ERK2), NFKB1, PTEN, TIMP2, TIMP3, TP53 (p53), VEGFA. |
|  |  |
| **Cell Cycle** | APC, BCL2, CASP3, CAV2, CCNA1, CCND1, CCND2, CDKN2A (P16INK4A), EGFR (ERBB1), IGF1, PPP2R1B, PTEN, PTGS1 (COX1), PTGS2 (COX2), TP53 (p53). |
|  |  |
| **Transcription Factors** | AR, ARNTL (BMAL1), CDKN2A (P16INK4A), CREB1, DAXX, EGR3, ERG, ETV1, FOXO1, MAX, MSX1, NFKB1, NKX3-1, NRIP1, RARB, RBM39, SOX4, SREBF1, SUPT7L, TP53 (p53). |
|  |  |
| **Fatty Acid Metabolism** | ACACA, CAMKK1, FASN, HMGCR, IGF1, PRKAB1, SREBF1, STK11 (LKB1). |
|  |  |
| **Other Prostate Cancer Genes** | MKI67, TMPRSS2. |
|  |  |

**Supplementary Table S8.** Gene list of the RT² Profiler Human Cancer Inflammation & Immunity Crosstalk PCR Array (#PAHS-181Z)

| **Gene categories** | **Gene symbols** |
| --- | --- |
|  |  |
| **Immune & Inflammatory Responses** | ***Immunostimulatory Factors*:** IFNG, IL12A, IL15, IL2, TNF.  ***Immunosuppressive Factors*:** CD274 (PD-L1), CSF2 (GM-CSF), CTLA4, CXCL12 (SDF1), CXCL5 (ENA-78, LIX), CXCL8 (IL8), IDO1 (IDO), IL10, IL13, IL4, MIF, NOS2 (iNOS), PDCD1 (PD1), PTGS2 (COX2), TGFB1, VEGFA.  ***Pro-Inflammatory Genes*:** CCL2 (MCP-1), CCL20 (MIP-3A), IFNG, IL12A, IL12B, IL17A, IL1A, IL1B, IL2, IL23A, IL6, PTGS2 (COX2), TLR4, TNF, VEGFA.  ***Anti-Inflammatory Genes*:** IL10, IL13, IL4, TGFB1.  ***Enzymatic Modulators of Inflammation & Immunity*:** AICDA (AID), GZMA, GZMB, IDO1 (IDO), NOS2 (iNOS), PTGS2 (COX2). |
|  |  |
| **Antigen Presentation** | HLA-A, HLA-B, HLA-C, MICA, MICB. |
|  |  |
| **Chemokines** | CCL18 (PARC), CCL2 (MCP-1), CCL20 (MIP-3A), CCL21 (MIP-2), CCL22 (MDC), CCL28, CCL4 (MIP-1B), CCL5 (RANTES), CXCL1 (GRO1, GROa, SCYB1), CXCL10 (INP10), CXCL11 (I-TAC, IP-9), CXCL12 (SDF1), CXCL2 (GRO2, GROb, SCYB2), CXCL5 (ENA-78, LIX), CXCL9 (MIG). |
|  |  |
| **Chemokine Receptors** | ACKR3, CCR1, CCR10, CCR2, CCR4, CCR7, CCR9, CXCR1, CXCR2, CXCR3, CXCR4, CXCR5. |
|  |  |
| **Interleukins** | CXCL8 (IL8), IL10, IL12A, IL12B, IL13, IL15, IL17A, IL1A, IL1B, IL2, IL23A, IL4, IL6. |
|  |  |
| **Other Cytokines** | KITLG (SCF), MIF, SPP1, TNF, TNFSF10 (TRAIL). |
|  |  |
| **Growth Factors & Receptors** | CSF1 (MCSF), CSF2 (GM-CSF), CSF3 (GCSF), EGF, EGFR (ERBB1), IGF1, TGFB1, VEGFA. |
|  |  |
| **Signal Transduction** | ***Interferon Signaling*:** GBP1, IFNG, IL6, IRF1.  ***Interferon-Responsive Genes*:** CCL2 (MCP-1), CCL5 (RANTES), CXCL10 (INP10), CXCL9 (MIG), GBP1, IRF1, MYD88, STAT1, TLR3, TNFSF10 (TRAIL).  ***NFκB Targets*:** BCL2L1 (BCLXL), CCL2 (MCP-1), CCL5 (RANTES), CSF1 (MCSF), CSF2 (GM-CSF), CSF3 (GCSF), CXCL8 (IL8), IFNG, TNF.  ***STAT Targets*:** CCL2 (MCP-1), CCL4 (MIP-1B), CCL5 (RANTES), CSF1 (MCSF), CSF2 (GM-CSF), CSF3 (GCSF), CXCL10 (INP10), CXCL11 (I-TAC, IP-9), CXCL12 (SDF1), CXCL8 (IL8), CXCL9 (MIG), IL10, IL17A, IL1B, IL23A, IL6, MYC.  ***Toll-Like Receptor Signaling*:** MYD88, TLR2, TLR3, TLR4.  ***Transcription Factors:*** FOXP3, HIF1A, IRF1, MYC, NFKB1, STAT1, STAT3, TP53 (p53). |
|  |  |
| **Apoptosis** | ***Pro-Apoptotic*:** FASLG (TNFSF6), TNF, TNFSF10 (TRAIL), TP53 (p53).  ***Anti-Apoptotic*:** BCL2, BCL2L1 (BCLXL), MYC, STAT3. |
|  |  |

**Supplementary Table S9.** Immunohistochemical analysis of inflammation and immunity genes and prostate cancer driver genes in wild type and IL30 gene transfected tumors

|  | **Human Tumor Xenografts** | | | |
| --- | --- | --- | --- | --- |
|  | **WT-PC3*** | **IL30-PC3** | **WT-DU145*** | **IL30-DU145** |
|  |  | | | |
| **Inflammation & Immunity Genes**^§^ |  |  |  |  |
| IL30 | 6.0 ± 0.5 | 18.0 ± 5.5^‡^ | 8.8 ± 1.7 | 19.2 ± 6.0^‡^ |
| IL12B | 4.9 ± 1.3 | 18.2 ± 5.2^‡^ | 5.7 ± 2.0 | 16.8 ± 4.0^‡^ |
| TNFα | 4.3 ± 0.7 | 8.6 ± 1.6^‡^ | 4.9 ± 1.0 | 10.0 ± 2.2^‡^ |
|  |  | | | |
| **Prostate Cancer Driver Genes**^§^ |  | | | |
| SHBG | 2.9 ± 0.6 | 8.4 ± 2.6^‡^ | 1.8 ± 0.6 | 14.4 ± 3.6^‡^ |
| LGALS4 | 3.2 ± 08 | 10.2 ± 2.5^‡^ | 3.9 ± 0.8 | 9.0 ± 3.3^‡^ |
|  | | | | |

* Results from EV-transfected tumors were comparable to results from wild type (WT) tumors.

^§^ Expression values of IL30, prostate cancer driver genes and inflammation and immunity genes are represented as the mean percentage ± SD of positively stained areas/total area of the examined fields (85 431.59 μm^2^) at ×400.

^‡^ Values significantly different (Student's t-test: p<0.01) from corresponding values in WT-PC3 and WT-DU145 tumors.

**Supplementary Table S10.** Morphometric evaluation of IL30 and immunoregulatory and prostate cancer driver gene expression in prostate cancer samples from patients of the validation cohort*

| **Pat. num.** | **Gleason score** | **IL30** | **IL12B** | **SHBG** | **LGALS4** | **GNRH1** | **NOS2** | **TNFA** | **CXCR5** |
| --- | --- | --- | --- | --- | --- | --- | --- | --- | --- |
| 1 | 7 | 40 | 12 | 38 | 14 | 24 | 12 | 40 | 48 |
| 2 | 6 | 12 | 17 | 56 | 16 | 38 | 35 | 19 | 33 |
| 3 | 7 | 34 | 19 | 50 | 49 | 31 | 78 | 71 | 83 |
| 4 | 9 | 83 | 86 | 88 | 66 | 24 | 22 | 47 | 27 |
| 5 | 5 | 30 | 39 | 21 | 38 | 17 | 26 | 48 | 26 |
| 6 | 7 | 31 | 46 | 29 | 32 | 16 | 47 | 15 | 28 |
| 7 | 7 | 38 | 13 | 46 | 19 | 47 | 24 | 35 | 28 |
| 8 | 5 | 10 | 34 | 13 | 77 | 40 | 22 | 37 | 22 |
| 9 | 6 | 34 | 21 | 15 | 59 | 29 | 36 | 21 | 16 |
| 10 | 7 | 18 | 41 | 37 | 42 | 35 | 17 | 21 | 36 |
| 11 | 9 | 68 | 65 | 82 | 54 | 61 | 64 | 68 | 53 |
| 12 | 7 | 90 | 92 | 93 | 60 | 91 | 77 | 81 | 87 |
| 13 | 2 | 45 | 95 | 36 | 11 | 39 | 76 | 94 | 71 |
| 14 | 7 | 53 | 34 | 60 | 41 | 44 | 25 | 38 | 38 |
| 15 | 3 | 49 | 12 | 33 | 37 | 31 | 39 | 32 | 35 |
| 16 | 3 | 11 | 33 | 18 | 51 | 27 | 23 | 32 | 36 |
| 17 | 7 | 55 | 16 | 53 | 76 | 69 | 15 | 43 | 43 |
| 18 | 5 | 48 | 60 | 25 | 15 | 24 | 53 | 65 | 58 |
| 19 | 6 | 17 | 42 | 32 | 11 | 25 | 28 | 30 | 39 |
| 20 | 7 | 65 | 51 | 80 | 60 | 52 | 52 | 59 | 50 |
| 21 | 8 | 62 | 67 | 84 | 50 | 42 | 68 | 68 | 70 |
| 22 | 4 | 28 | 4 | 32 | 21 | 40 | 26 | 30 | 30 |
| 23 | 9 | 49 | 67 | 34 | 20 | 42 | 52 | 55 | 68 |
| 24 | 4 | 49 | 70 | 23 | 28 | 30 | 67 | 59 | 57 |
| 25 | 9 | 61 | 67 | 93 | 62 | 81 | 67 | 63 | 60 |
| 26 | 7 | 26 | 60 | 39 | 30 | 15 | 54 | 52 | 55 |
| 27 | 7 | 49 | 68 | 54 | 26 | 24 | 70 | 57 | 66 |
| 28 | 7 | 84 | 90 | 87 | 51 | 60 | 16 | 25 | 82 |
| 29 | 7 | 44 | 25 | 16 | 39 | 44 | 46 | 15 | 15 |
| 30 | 9 | 89 | 73 | 75 | 52 | 85 | 51 | 50 | 69 |
| 31 | 7 | 30 | 5 | 29 | 27 | 23 | 43 | 32 | 36 |
| 32 | 7 | 35 | 28 | 40 | 45 | 23 | 30 | 19 | 32 |
| 33 | 7 | 47 | 42 | 12 | 48 | 17 | 33 | 18 | 35 |
| 34 | 8 | 66 | 60 | 70 | 75 | 33 | 59 | 55 | 51 |
| 35 | 5 | 38 | 14 | 22 | 67 | 17 | 69 | 59 | 67 |
| 36 | 9 | 51 | 70 | 66 | 78 | 50 | 58 | 57 | 57 |
| 37 | 5 | 16 | 32 | 16 | 54 | 46 | 35 | 50 | 55 |
| 38 | 4 | 47 | 96 | 41 | 52 | 15 | 71 | 93 | 43 |
| 39 | 8 | 46 | 75 | 25 | 29 | 46 | 55 | 54 | 68 |
| 40 | 7 | 23 | 32 | 32 | 48 | 34 | 16 | 27 | 33 |
| 41 | 7 | 19 | 8 | 21 | 58 | 36 | 26 | 42 | 25 |
| 42 | 7 | 23 | 47 | 46 | 30 | 47 | 54 | 53 | 67 |
| 43 | 7 | 59 | 65 | 90 | 68 | 43 | 57 | 61 | 57 |
| 44 | 3 | 10 | 29 | 45 | 82 | 20 | 13 | 24 | 67 |
| 45 | 7 | 14 | 41 | 52 | 49 | 17 | 67 | 55 | 34 |
| 46 | 8 | 55 | 15 | 71 | 80 | 65 | 27 | 36 | 21 |
| 47 | 8 | 55 | 18 | 52 | 66 | 87 | 41 | 25 | 25 |
| 48 | 3 | 21 | 11 | 18 | 49 | 26 | 13 | 42 | 36 |
| 49 | 7 | 28 | 20 | 52 | 25 | 47 | 10 | 32 | 20 |
| 50 | 10 | 85 | 83 | 86 | 58 | 21 | 73 | 76 | 88 |
| 51 | 8 | 90 | 93 | 90 | 62 | 80 | 76 | 90 | 14 |
| 52 | 7 | 57 | 70 | 67 | 79 | 86 | 54 | 65 | 69 |
| 53 | 7 | 15 | 16 | 23 | 16 | 29 | 66 | 70 | 92 |
| 54 | 7 | 43 | 5 | 20 | 56 | 35 | 26 | 18 | 20 |
| 55 | 7 | 28 | 7 | 53 | 57 | 43 | 12 | 16 | 34 |
| 56 | 7 | 27 | 40 | 32 | 57 | 31 | 41 | 42 | 21 |
| 57 | 7 | 22 | 13 | 22 | 56 | 44 | 49 | 41 | 23 |
| 58 | 7 | 27 | 33 | 24 | 80 | 39 | 26 | 46 | 24 |
| 59 | 7 | 48 | 46 | 11 | 31 | 39 | 34 | 17 | 32 |
| 60 | 7 | 42 | 12 | 51 | 50 | 43 | 19 | 50 | 55 |
| 61 | 7 | 41 | 13 | 40 | 11 | 35 | 84 | 72 | 80 |
| 62 | 8 | 70 | 78 | 72 | 59 | 51 | 88 | 76 | 73 |
| 63 | 8 | 43 | 43 | 19 | 29 | 27 | 16 | 24 | 26 |
| 64 | 7 | 70 | 43 | 94 | 48 | 41 | 43 | 22 | 77 |
| 65 | 3 | 23 | 43 | 20 | 19 | 24 | 49 | 35 | 31 |
| 66 | 6 | 48 | 49 | 16 | 47 | 27 | 57 | 61 | 50 |
| 67 | 6 | 41 | 35 | 37 | 45 | 32 | 58 | 68 | 51 |
| 68 | 5 | 13 | 14 | 16 | 15 | 82 | 22 | 49 | 57 |
| 69 | 7 | 37 | 32 | 20 | 42 | 38 | 28 | 38 | 45 |
| 70 | 6 | 39 | 26 | 48 | 27 | 29 | 63 | 54 | 50 |
| 71 | 4 | 14 | 35 | 56 | 26 | 30 | 40 | 30 | 21 |
| 72 | 7 | 13 | 34 | 52 | 25 | 78 | 45 | 22 | 26 |
| 73 | 7 | 39 | 39 | 42 | 31 | 15 | 26 | 20 | 25 |
| 74 | 7 | 27 | 60 | 42 | 12 | 48 | 69 | 67 | 61 |
| 75 | 5 | 25 | 56 | 22 | 51 | 47 | 67 | 63 | 52 |
| 76 | 7 | 30 | 15 | 21 | 48 | 28 | 16 | 49 | 56 |
| 77 | 2 | 14 | 45 | 31 | 41 | 27 | 34 | 42 | 42 |
| 78 | 7 | 22 | 5 | 56 | 41 | 29 | 14 | 35 | 46 |
| 79 | 7 | 20 | 32 | 45 | 19 | 38 | 22 | 45 | 23 |
| 80 | 7 | 24 | 64 | 59 | 60 | 24 | 64 | 70 | 20 |

* Expression values of immunoregulatory genes and prostate cancer driver genes are represented as the mean percentage of the positively stained area/total area of the examined fields.
